# The evolution of an ancient technology

**DOI:** 10.1098/rsos.170208

**Published:** 2017-05-31

**Authors:** Christopher D. Buckley, Eric Boudot

**Affiliations:** 1 College Farm, Banbury OX15 5TB, UK; 2 École Pratique des Hautes Études, Paris, France

**Keywords:** cultural evolution, cultural transmission, phylogenetic analysis, cultural phylogeny, evolution of technology, traditional weaving

## Abstract

We investigate pattern and process in the transmission of traditional weaving cultures in East and Southeast Asia. Our investigation covers a range of scales, from the experiences of individual weavers (‘micro’) to the broad-scale patterns of loom technologies across the region (‘macro’). Using published sources, we build an empirical model of cultural transmission (encompassing individual weavers, the household and the community), focussing on where cultural information resides and how it is replicated and how transmission errors are detected and eliminated. We compare this model with macro-level outcomes in the form of a new dataset of weaving loom technologies across a broad area of East and Southeast Asia. The lineages of technologies that we have uncovered display evidence for branching, hybridization (reticulation), stasis in some lineages, rapid change in others and the coexistence of both simple and complex forms. There are some striking parallels with biological evolution and information theory. There is sufficient detail and resolution in our findings to enable us to begin to critique theoretical models and assumptions that have been produced during the last few decades to describe the evolution of culture.

## 1. Introduction

One of the challenges of investigating culture is to combine understanding of the micro-level processes by which it is transmitted with understanding of the broad-scale patterns that this transmission generates. With this in mind, we report a new study that examines an aspect of traditional culture in a systematic way across a range of scales. Our chosen area of study is traditional weaving cultures in East and Southeast Asia. Weaving is predominantly a home-based activity that plays a central role in life and customs in this region. We chose to examine loom technology in particular, because it is culturally specific with large differences between neighbouring cultures, because of its richness and complexity in the East Asia region and because it is transmitted conservatively within cultural groups. There is also intrinsic interest in how complex technologies (as an aspect of complex culture) develop and how they are sustained. In the settings that we examined, the written word plays little or no role in the transmission of traditional weaving, so the results can shed light on how this process occurs in pre-literate societies.

Considered in the most general sense, technology consists of knowledge about how to modify our environment, passed from one generation to the next. At a popular level, ‘technology’ is synonymous with ‘progress’, particularly because of the rapid advances that have occurred in some cultures during the past few centuries. But technological change has a much longer history, and recent developments may not be a good guide to the broad span of human history. An understanding of how technology arises and the circumstances under which it becomes complex (and those under which it does not) remains a central question in human culture.

Technological change was investigated by Basalla [1], who noted that it is a gradual process and concluded that it is ‘analogous’ to biological evolution. More recently, it has been reviewed by Boyd *et al*. [2], Mesoudi *et al.* [3] and Shennan [4]. These authors also concluded that technology ‘evolves’ based upon general observations (‘stylized facts’), theoretical models and a limited number of case studies. The models, which include both analytical and agent-based computational approaches, suggest that cumulative cultural evolution is possible under a wide range of evolutionary models and starting conditions. Shennan and Steele [5] reviewed ethnographic literature on craft and tool-making traditions and found that such traditions tend to be transmitted in a ‘vertical’ fashion, predominantly from father to son or mother to daughter. There is broad agreement, however, on the need for more empirical studies, to identify actual conditions and processes.

For participants, the significance of weaving extends beyond practical concerns and is linked with other aspects of complex culture, including ethnic identity (where it ranks alongside language/dialect in importance), displays of wealth and status and customs related to rites of passage (see Maxwell [6], Hunt-Kahlenberg and Barnes [7] for reviews of these topics). The products of these traditions include some spectacular and complex textiles that play an important role in rituals related to marriage in particular. Studies on weaving traditions, focusing on the lineages of Iranian tribal decorative motifs, by Tehrani and co-workers [8–11], and by the present authors on woven ikat motifs in Southeast Asia [12] and the transmission of weaving cultures and looms in Southwest China [13] and East Nusa Tenggara [14] have shown that weaving practices are transmitted from generation to generation in a conservative manner, giving rise to distinct lineages that persist over time.

The questions we set out to answer in this study are the following:

1. what information characterizes a weaving tradition, and where does it reside?
2. how is this information passed from generation to generation?
3. what are the macro-level consequences of these micro-level transmission processes, for example in the relationships between loom designs used by different groups?
4. how and under what circumstances do complex technologies emerge? and
5. under what circumstances are technical inventions transmitted from one group to another?

The changes that have occurred in looms are the sum of the modifications and inventions made by individuals over a long period. Ideally we would like to have biographies of the individuals involved, but in most cases such information does not exist. Instead, we make use of data relating to present-day looms, and we use a phylogenetic method to reconstruct the pathways by which technologies developed. Our justification for using this method is based on our analysis of transmission processes. Similar systematic approaches have been applied to understanding a range of cultural phenomena, including languages [15,16], societal structure [17], farming practices [18], the development of stone tools [19,20], the evolution of folktales [21,22] traditional longhouse architecture [23], basket-making traditions [24] and hunter–gatherer technologies [25]. The results of these studies reveal a variety of processes and patterns of cultural change, including both intra-community and inter-community transmission.

Weaving consists of making a textile by interlacing flexible yarns (warp and weft), usually at right angles. A loom is a device that facilitates this by tensioning warp yarn between two supports, allowing the weft to be inserted with the fingers, with a shuttle or with a hook. The simplest looms in our study consist of two beams over which the warp is stretched, with a few additional rods, though many elaborations are possible that speed up the process, allow a longer or wider cloth to be woven, or facilitate adding decoration to the cloth during the weaving process. The looms we examined in our study are oriented horizontally, or at a slight angle to the horizontal, and are used to weave wide panels of cloth (as opposed to narrow bands or mats). In many of them, the warp is tensioned by the weaver's own body.

**Figure 1.**
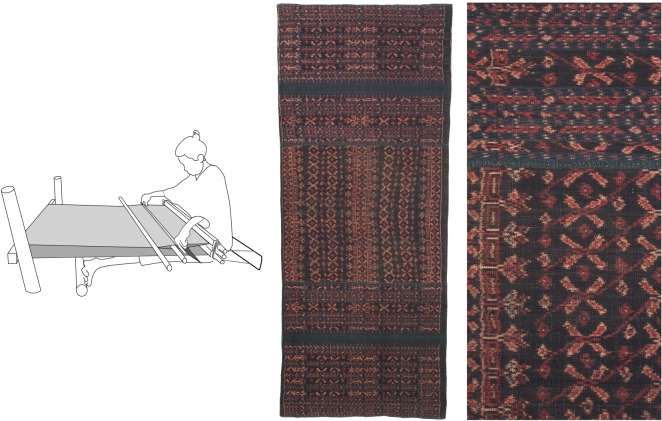
A simple, ground-level loom from Nggela in Flores (Ende Regency), used to weave ikat-decorated cotton sarongs such as the one shown to the right of the loom (whole sarong, detail of ikat decoration).

**Figure 2.**
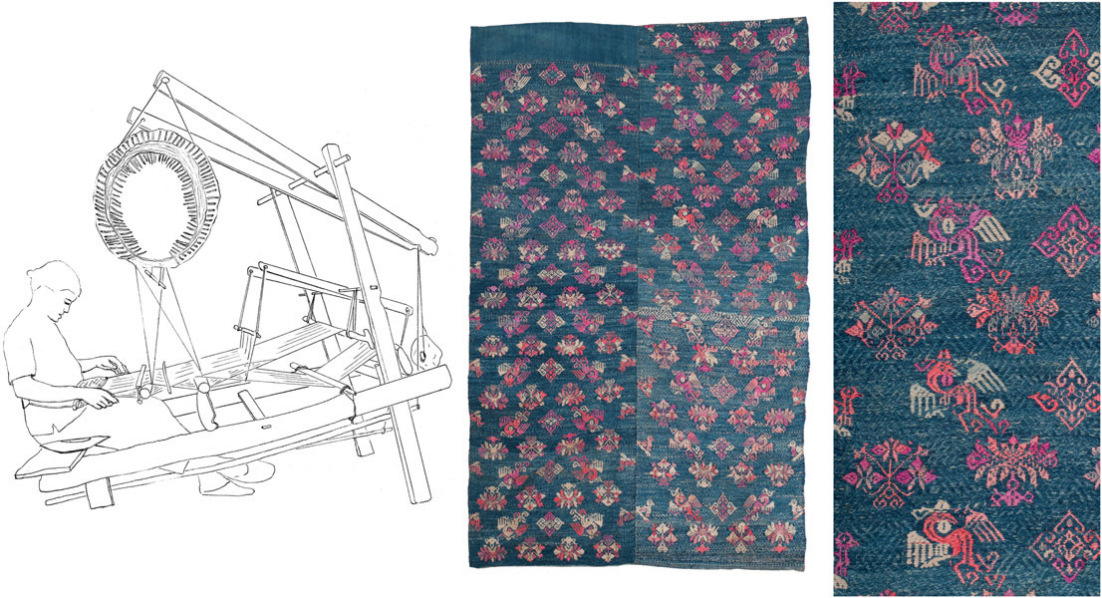
A loom used by Maonan weavers in Huanjiang county, Guangxi province, China. One of the most complex looms in our study, it incorporates a ‘programmed’ textile pattern, saved in the form of bamboo rods embedded in cords around a bamboo drum (near to the weaver and just above her head). It is used to weave bedcovers such as the one shown to the right of the loom, which consists of two woven strips sewn together. The detail at the far right shows part of the repeating motifs in silk supplementary weft on an indigo cotton ground, each repeat corresponding to one rotation of the pattern drum.

There is a very wide range of loom designs in our study region: the variation in complexity is illustrated by the two looms in figures 1 and 2. The first of these is a simple ground-level, body-tensioned loom from the island of Flores. Looms of this general type are found across the eastern Indonesian archipelago, in the Philippines, in Southeast Asia and in the Himalayan foothills. The second loom is an example of one of the more complex types of loom, of which there are also many different types, which are found on the East Asian mainland, in Malaysia and in the western islands of Indonesia. This particular loom, used by Maonan people in Guizhou province, China, incorporates a pattern-recording system consisting of a large bamboo drum with a web of cords around it. Bamboo sticks embedded in this web record a sequence of warp lifts for making complex designs using silk supplementary weft on a cotton ground. At first glance this loom looks completely different from the simple ground-level loom, but these two looms share some important features, including horizontal orientation, body-tensioning (via a strap around the weaver's back), a warp beam that is lodged behind two posts (rather than being fixed permanently in the frame), an arrangement for making ground-weave that consists of a single heddle and a rod that retains a permanent opening in the warp and the use of a wooden beater with a sharp edge to beat-in the weft. Both kinds of loom are used to make elaborately patterned textiles that are important in marriage rituals and as markers of status, examples of which are also shown in the figures.

A primer on loom weaving technology and terminology is provided in the electronic supplementary material, S1.

## 2. Material and methods

As a first step, we assembled data on cultural transmission processes from published accounts of weaving cultures in the East Asia region, comprising two area surveys [13,14] and four studies of individual cultures [26–29]. The first two studies (by the present authors) examined cultural transmission processes among weaving communities in Southwest China (SWC) and East Nusa Tenggara (ENT). Findings from these studies are summarized in table 1. The other works are in-depth studies on individual weaving communities in the Himalayan region, Sumatra and in ENT that provide complementary insights into the detailed processes by which weaving skills are transmitted. We analysed these data by identifying and classifying where weaving-related information (in the most general sense of the term) resides and in what forms, and the processes by which this information is maintained and transmitted.

**Table 1.** Summary of weaver's experiences in ‘learning to weave’ from published ethnographic surveys in Southwest China [13] and East Nusa Tenggara [14].

			Southwest China (Guizhou, Guangxi, West Hunan)	East Nusa Tenggara (Eastern Flores, Solor and Lembata, Indonesia)	combined totals
	*number of distinct weaving traditions*		*18*	*22*	*40*
	*number of weavers interviewed*		*27*	*26*	*53*
	question:	categorization of responses:			
	weaver's origin	*same dialect and weaving tradition*	27	25	53
		*different dialect and weaving tradition*	—	1	1
	patrilocal/ matrilocal		all patrilocal	all patrilocal	
	weaver's average age (estimated)		50	58	54
	age when began to learn to weave (average)		13	16	15
	age when began to learn to weave (range)		5–16	10–20	5–20
	which person(s) did you learn weaving from	*mother*	27	17	44
		*grandmother*	1	1	2
		*aunt*	—	1	1
		*other woman from the same village*	—	1	1
		*no-one/'self taught'*	—	6	6
	marriage-related customs		all groups weave a number of standardized items before marriage, for presentation and display at courtship events and at the wedding. These items are woven by young women before marriage, assisted by older female relatives (primarily the mother). These items are retained by the bride after marriage, as her personal property. Some weavers also make items for sale to non-weaving brides	weavers make bridewealth exchange gifts between families, as well as daily-use items. Bridewealth items are standardized by tradition. Some weavers weave items of this type (as well as daily-use items) for sale to non-weaving families or neighbouring non-weaving groups. Bridewealth items are generally woven by older, experienced weavers	
	templates used for recording textile designs (if any)	*old textile*	8	(most claim to rely on memory, but also seem to refer to old textiles from time to time)	8
		*embroidered sampler*	2 (Miao groups)	—	2
		*complex pattern heddle with embedded pattern rods*	5 (Tai-Kadai groups)	—	5
		*patterns are learned and memorized by the weaver (wholly or in part)*	11	26	37
	domesticates (fibre sources)	*silk moth*	9	—	9
		*cotton*	15	all formerly grew cotton, few do nowadays	15
		*hemp*	2	—	2
		*ramie*	1	—	1
		*wool*	3 (hemp and ramie cultivation appear to have been more common in the past)	—	3
	loom type		frame loom with back tensioning and foot-operated heddle-raising mechanism	simple, ground-level back-tension loom	
	how is the loom made		looms are kept by families and are associated with particular houses, where they remain. When a new loom is required, a local carpenter or a male relative with carpentry skills is called in to copy an existing loom. In the case of the Jianghua Yao, looms are passed by mother to daughter or daughter-in-law and are regarded as belonging to individuals	looms are passed from mother to daughter. Replacement components are made by male relatives when needed. The weaver's sword-beater is the critical component, made from a hardwood and requiring careful shaping. Other components can be made ad hoc from materials lying around	

**Figure 3.**
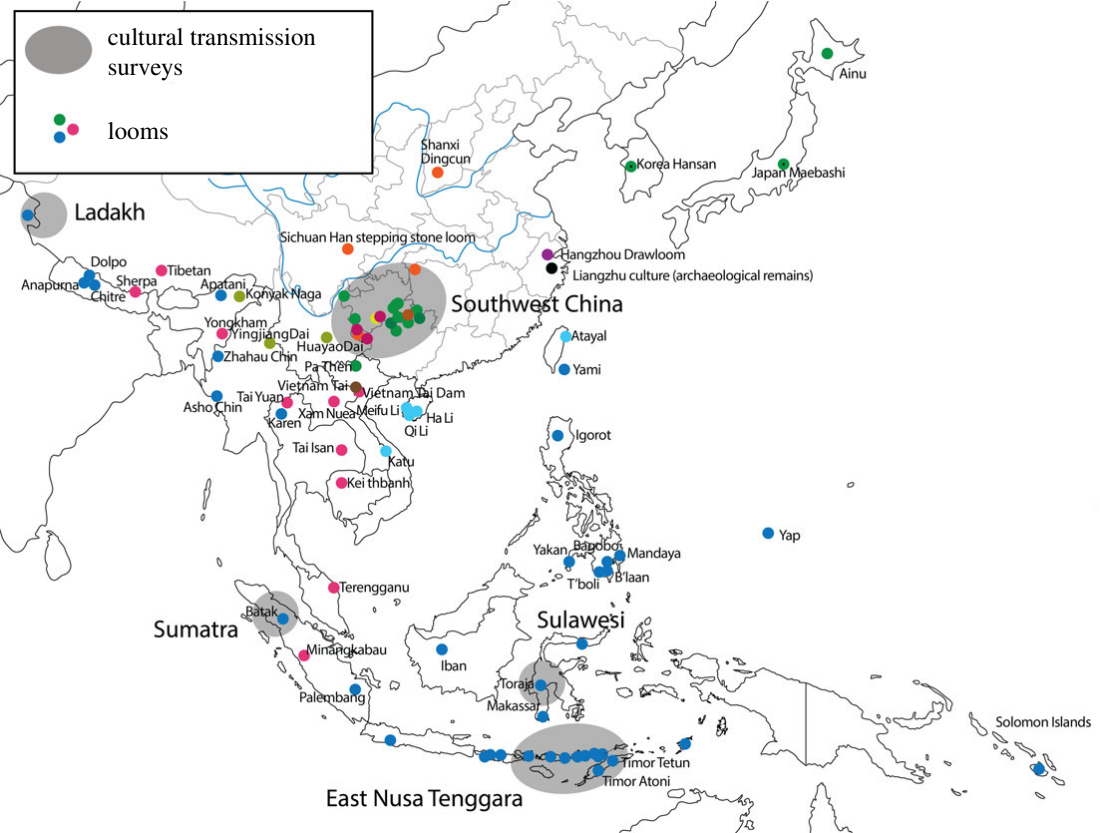
Locations of weaving traditions studied in this paper. Grey ellipses show the locations of cultural transmission studies [13,14,26–29]. Dots show the locations of looms in our survey of loom technologies.

For the second part of our study, we report a new survey of loom designs from 85 locations, chosen to represent the widest possible variety of designs, including looms ranging from simple ground-level devices consisting of a dozen or so rods, to complex frame looms with hundreds of individual parts (such as the Maonan loom in figure 2). The looms are from a geographical region encompassing Southeast Asia, East Asia, Island Southeast Asia and parts of Oceania (figure 3). Most of the looms are used in domestic settings. Three looms in our study (the stepping stone loom, and the greater and lesser drawlooms) were used in commercial workshop settings until the early part of the twentieth century, when they were replaced by imported Jacquard looms. The domestic looms have continued to be used to the present day, though traditional weaving is presently in decline in most areas.

Our data sources include examples of looms held in museum collections, public displays of weaving and published sources. These sources are listed in the electronic supplementary material, S2. We also reviewed published accounts on archaeological remains of looms in the region, and we include the remains of the oldest clearly identifiable loom in our dataset. This is a body-tensioned loom found in a tomb from the Liangzhu culture (BCE 3400–2250), in Zhejiang province, China [30].

We analysed the looms in terms of their components, interconnections and how each component is used. A master list of around 400 distinct features (characters) was identified, of which 290 were found to be phylogenetically informative (listed in the electronic supplementary material, S3). Each feature was marked as present (1) or absent (0) for each loom. The results are contained in a table in Nexus format (electronic supplementary material, S4). To our knowledge, this is the largest database of its kind that relates to material culture.

## 3. Summary and analysis of cultural transmission processes based on published accounts

All six of the studies that we drew upon for our analysis report the same basic pattern of transmission: weaving-related practices are passed inter-generationally (‘vertically’), primarily from mother to daughter, and to a lesser extent between other female weavers within the community. This transmission takes place within the dialect community, which in most cases consists of a group of nearby villages sharing the same dialect, customs and textile/dress traditions. There are no reported examples of the transmission of weaving-related knowledge occurring between communities (though there is indirect evidence that this has occurred in the past, as we discuss below), and we conclude that this process is comparatively rare.

Within the overall pattern of mother-to-daughter transmission, there is some variation in the detail of how transmission occurs. In SWC, weaving takes place mainly in the home and is almost entirely a ‘family’ affair. In ENT, weaving tends to be done outdoors in the village and is carried out by small groups of women who habitually weave together. Transmission of the weaving technique is still mainly from mother to daughter in ENT, but young weavers have the opportunity to observe weavers from other households during their apprenticeship.

Within communities, the main vector that moves cultural information between households is marriage, since weavers tend to move from the parental household to their spouse's household or found a new household when they marry (often doing both, sequentially). Marriage within the dialect community is the norm in all of the communities. Households are often linked by long-standing marriage alliances, and marriage itself is attended by public rituals, including the display of textiles (SWC) and/or the exchange of textiles (ENT). Significant barriers exist to marriage outside of the dialect community, and this is correspondingly rare, despite recent social changes that are tending to break down such barriers. Marriage, therefore, circulates weaving-related practices between households within a community, but rarely outside of it.

Loom designs and weaving techniques are reported to be uniform within communities, with few or no differences in technique between households. By contrast, substantial differences in looms and technique are evident between communities in SWC, and to a lesser extent in ENT. As regards motifs and clothing styles, there are variations both between individual weavers and between households, but these are mainly attributable to selection from a well-defined ‘repertoire’. For example, no two ceremonial sarongs from the Atadei area of Lembata are alike, but the style of an Atadei sarong is nevertheless recognizable and different from the sarongs produced in other parts of Lembata.

In all of the weaving communities, it is apparent that novice weavers have few choices available to them in questions of ‘how to learn’ and ‘who to learn from’, particularly where technique is concerned. These questions are determined by the traditional practices of the community. In all of the communities, the learning process consists of a lengthy apprenticeship during which the novice observes and then learns to replicate the various steps related to fibre and yarn preparation, dyeing and weaving. Some parts of the process, particularly the more complex ones such as setting up a new warp on a loom, have ‘ritual’ aspects that are integral to the process, with codified procedures including (for example) the selection of an auspicious day, making offerings to spirits or ancestors and the observance of taboos. Structured ‘teaching’ (defined as an activity where the sole purpose is to instruct a novice) is absent, but older weavers take care to correct errors that they notice younger weavers making.

There are differences in the degree to which various aspects of weaving culture are constrained by tradition. ‘Process’-related questions, particularly complex activities such as the preparation of dye baths, the warping of a loom and the form of the loom itself are the most conservative (highly constrained). The construction and layout of ceremonial textiles are also very conservative, with little or no opportunity for the novice to deviate from tradition. In other aspects, however, for example in the arrangement of motifs and the choice of colours, the novice weaver has some freedom to experiment. Some kinds of textile also offer more freedom: in ENT, daily-use sarongs are allowed more variation in terms of materials, colours and motifs than ceremonial sarongs.

Basic skills with a loom can be learned in a few months, but it takes longer to learn the full range of patterns and techniques. The ‘apprenticeship’ relationship can persist for a long period, often for many years. The master–novice relationship is not purely about learning to weave, but reflects the social structure and hierarchies of the community and defines the roles of the participants. Useful (and in some cases economically valuable) skills are passed from an older generation to a younger one, in return for help with routine tasks (such as yarn preparation and assistance with warping the loom) and the novice weaver's commitment to defer to her teacher and follow the community's traditions precisely.

A detailed summary of data on cultural transmission from the monographs on individual cultures [26–29] is included in the electronic supplementary material, S1. Looking beyond the East Asia region, similar dynamics were found, for example, by Tehrani and Collard [9] in their study of the intergenerational transmission of Iranian tribal weaving. Skills and motifs are transmitted mainly from mother to daughter and to a lesser extent between peers and more distant relatives within the tribal group. Barriers to intermarriage between groups tend to keep such information within the tribal group.

## 4. Classification of transmission processes

Considering the findings from the five studies discussed above, we classify the information that constitutes a weaving tradition into four distinct types:

1. the skills and knowledge of weavers;
2. tools (looms and other equipment);
3. templates (heirloom textiles, pattern samplers, pattern storage devices used on some looms); and
4. domesticates (plants and animals, used as sources of fibres and dyes).

The first type of information resides in the weaver's own head. The other three types, which are equally important, are embodied in physical formats that must be maintained and replicated. Looms and pattern templates are replaced from time to time as they wear out, while domesticates require replanting and husbandry. We include domesticates under the heading of ‘information’ since their genotypes embody the results of selective breeding (conscious or unconscious) over an extended period.

The novice weaver learns about the loom and the craft of weaving by learning ‘procedures’: sets of actions that are carried out in a predetermined manner and sequence. It is not necessary for a novice weaver to have a general understanding of how the loom works (a ‘theory of the loom’) in order to begin weaving, though experienced weavers do develop a sophisticated understanding of their craft, over time. In the case of the complex frame looms and patterning devices used in SWC, weavers are unable to recreate these devices from memory, and only a small proportion of talented weavers appear to understand in detail how they work. A carpenter can help a weaver to make a copy of a loom, but only if an existing model is available. For example, in one village in Guangxi where a particularly complex loom with a pattern-recording system is employed, only one weaver could be found who had the skill necessary to construct a patterning system for a new motif [13]. It appears that the level of skill needed to modify or renew a patterning system has always been relatively rare, with most households relying on hand-me-down pattern systems that have been in use for several generations. A similar situation is found in some Tai weaving villages in the Lao-Vietnam border region that also use complex patterning systems. In these villages, weaving is economically important, and there is some task specialization. The making of new patterning systems is usually undertaken by one or two households in each village that specialize in this work.

## 5. An empirical model for the transmission of weaving culture

**Figure 4.**
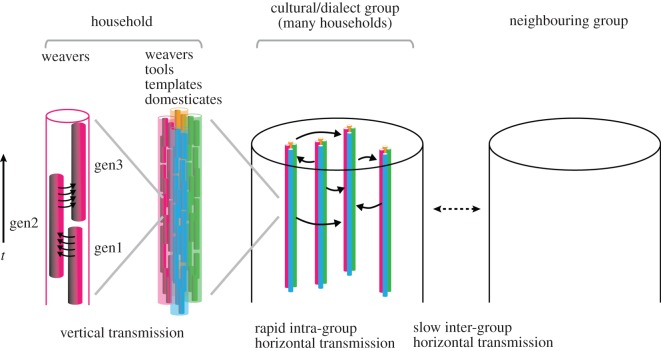
Schematic diagram summarizing cultural transmission processes. In this diagram, the vertical axis indicates time, and the horizontal axis generalized separation in space. The key information of weaving culture resides at the household level, in four forms: weaver's knowledge, tools, templates and domesticates. Weaving knowledge is passed between generations (gen1, gen2 and gen2), and tools, templates and domesticates are similarly maintained by overlapping generations. A group of households speaking the same dialect and sharing the same culture constitutes the cultural/dialect group (corresponding to a single taxon in our study). Within this group, weaving culture is exchanged via intermarriage, but exchange with neighbouring cultures is limited by barriers to intermarriage and cultural taboos.

Based on the foregoing, we construct an empirical model of the components of a weaving tradition and the transmission processes. This is summarized schematically in figure 4.

As noted, intermarriage seems to be the main vector by which weaving culture moves ‘horizontally’, intra-community, but information may also be shared via social contacts between weaving households, and during communal weaving activities (particularly in ENT). Conversely, exchange and intermarriage with neighbouring groups with different traditions is inhibited by ‘active barriers’ (community social norms) and by ‘passive barriers’ (such as a lack of appropriate bridewealth goods for ritual exchanges).

From the point of view of information flow (both ‘vertically’ in time, and ‘horizontally’ in space), the key features of this model are as follows:

1. vertical transmission of cultural information within households (‘unbiased transmission’) as the dominant mode;
2. horizontal exchange of cultural information between households of the same community as a secondary mode; and
3. a barrier to the horizontal flow of cultural information between different dialect communities.

Most of the focus of previous work on the transmission of culture has been on the first of these (vertical versus horizontal transmission), but in fact all three of these features are necessary for the formation of distinct and long-lived ‘cultural taxa’. Horizontal exchange of information between households (within taxon) is essential if the taxon is to be internally homogeneous: without this process, households would eventually evolve distinct traditions by a process of cultural drift. Barriers to information flow between taxa are necessary for taxa to remain distinct. This barrier is not completely impervious, however, as our findings related to looms demonstrate (discussed below).

The outstanding characteristics of the transmission processes are a lengthy apprenticeship that encourages ‘over-learning’, the orientation of older weavers towards detecting and correcting errors (deviations from tradition practice) and the codification of complex tasks into ritualized procedures. All of these features tend to increase the fidelity of transmission and discourage innovation.

## 6. Results and analysis of loom technology survey

Our review of micro-level processes suggests that loom designs are carefully conserved within dialect/weaving communities with barriers to sharing with neighbouring communities. This suggests that looms will form distinct lineages that persist over time, and that it may therefore be possible to describe the relationships between them with a tree-like model, at least to a first approximation. To test this hypothesis, we carried out a phylogenetic analysis, using the Bayesian Markov chain Monte Carlo method implemented in Mrbayes [31]. This method searches for trees that can reproduce the data, estimating the posterior probability of the trees and sampling them (after a ‘burn-in’ period) according to their probability (details in the electronic supplementary material, S1). We rooted the trees using the archaeological remains of the oldest known simple loom, from the Liangzhu culture. We assessed the goodness-of-fit by using a standard measure (retention index, RI), and by looking for conflicting signals in the data that might indicate that other types of (non-tree-like) processes may be operating. The RI is a measure of the amount of homoplasy in the data (shared character states that are owing to causes other than common ancestry): we chose this index in preference to the other commonly used measure, the consistency index, because the RI is independent of the number of taxa in the dataset, whereas the consistency index tends to generate lower scores for datasets with more taxa.

**Figure 5.**
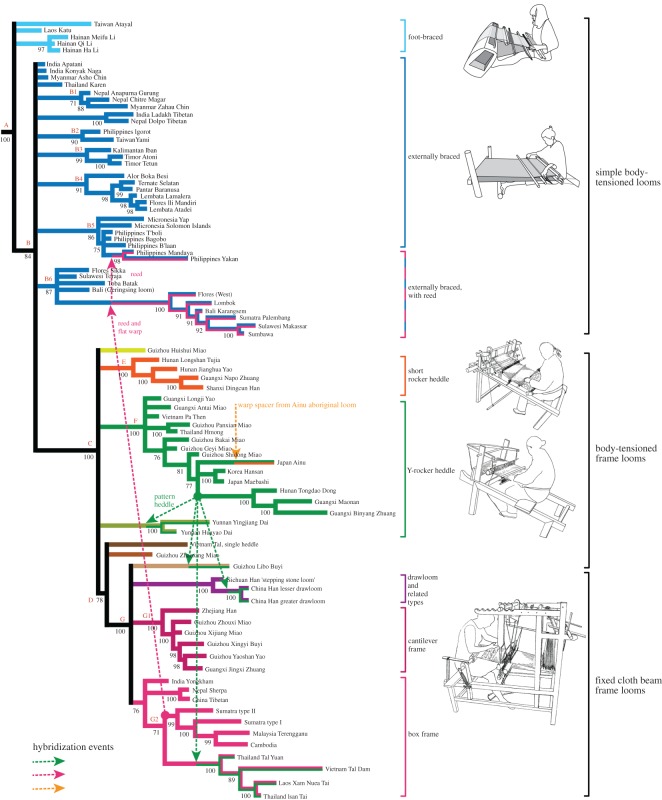
70% consensus tree, summarizing the results of the Bayesian phylogenetic analysis of the looms survey data. Colours and brackets indicate the main clades. Examples of actual looms are shown on the right to illustrate the structural features of the clades. Hybridization events (horizontal exchange of technologies) are shown by dotted lines linking lineages. Percentages of trees that contributed to each node are shown beside the nodes. Branch lengths indicate the approximate number of character changes in each lineage (not time depths).

The output of the Bayesian phylogenetic analysis consists of a set of trees, which we have summarized as a 70% consensus tree (figure 5). The RI for this tree is 0.7, which is towards the upper end of the range typically found for datasets that include a phylogenetic ‘signal’ [32]. This implies that a branching, tree-like model can reproduce the data reasonably well. This value can be compared with the RI of 0.5 obtained for lineages constructed using ikat decorative motifs for weaving traditions in ENT [12], suggesting that loom designs are transmitted more conservatively than the group of motifs in that study.

**Figure 6.**
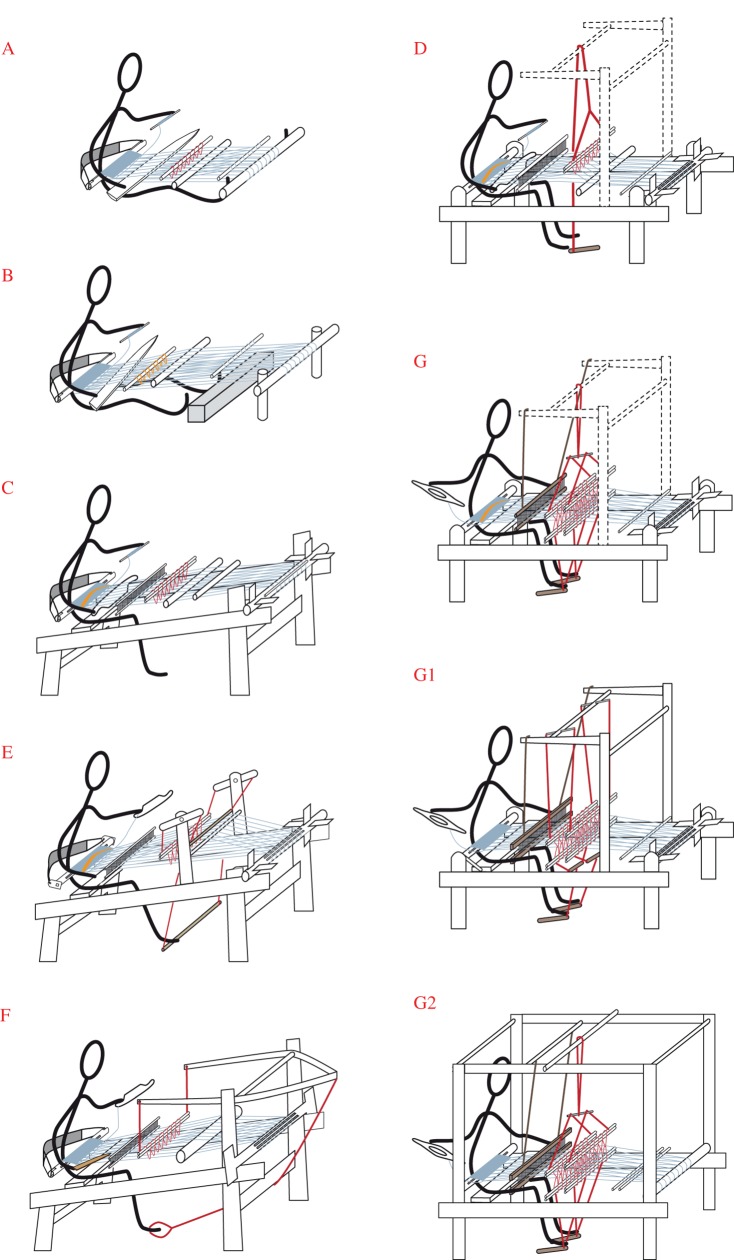
A-G2. Reconstruction of ancestral states on key nodes on the 70% consensus tree (figure 3). Ancestral states were reconstructed in Mesquite using a maximum-likelihood model.

As a second step, we computed the ancestral states at every node in the consensus tree (listed in the electronic supplementary material, file S5). We found that the reconstructed ancestral forms (figure 6) are all functional looms, which is an important indication that our model is a realistic one. From a weaving-technology standpoint, the resulting branching diagram is a model for loom evolution, with gradual, stepwise changes, from the late Neolithic period (the date of the earliest known loom, used to root the tree) to the present day. An account of the evolution of loom technologies implied by this model is included in the electronic supplementary material, S1.

The phylogenetic analysis provides sequences for loom innovations, but does not provide dates for these events, aside from the external reference point of the oldest known loom. Different characters appear to change at different rates, so there is no equivalent of a ‘molecular clock’ in loom evolution. Estimates of the time depths of some inventions can be inferred by comparison with archaeological and textual data, a topic that we have discussed elsewhere [13].

**Figure 7.**
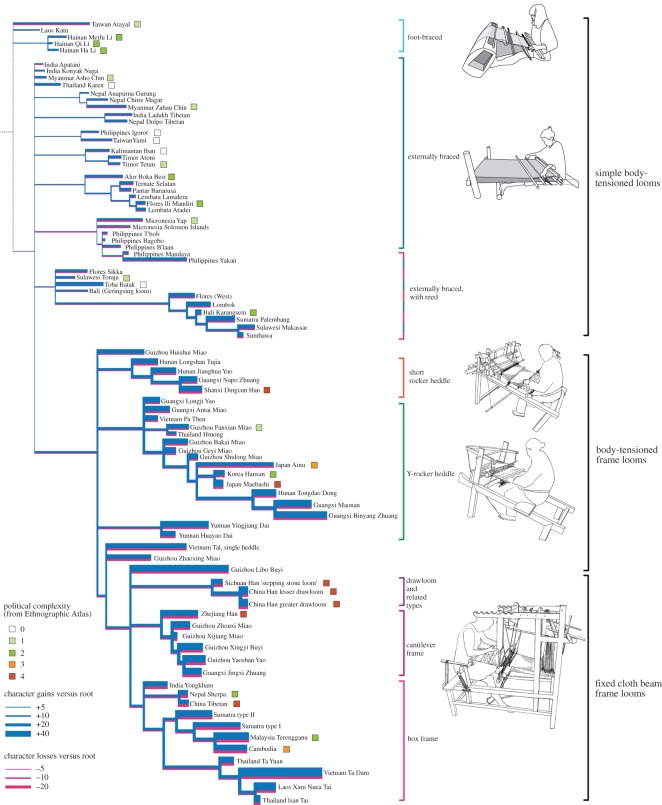
Seventy per cent consensus tree with character gains and losses superimposed, indicated by the widths of the blue and red bars (showing cumulative gains and losses, respectively) for each lineage.

As a third step, we examined the gain and loss of characters in each lineage, to assess where and how complexity developed. The numerical results of this analysis are shown superimposed on the lineages in figure 7. The results show that some lineages experienced relatively few changes over time (stasis), whereas others generated complex forms with many novel features, showing both gains and losses of characters.

**Figure 8.**
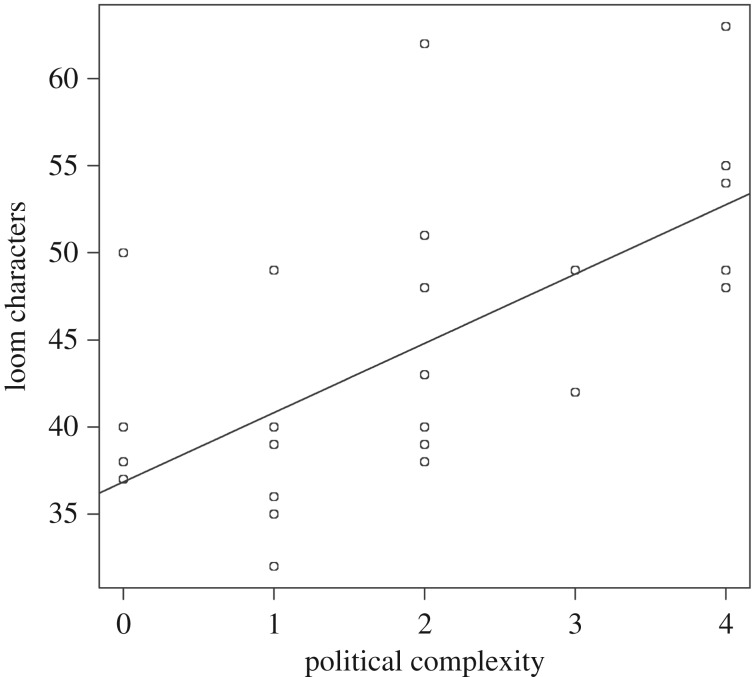
Linear regression of loom complexity (number of characters) versus political complexity of the associated culture (number of hierarchical levels in society, data from the Ethnographic Atlas). *R*^2^ = 0.42.

**Figure 9.**
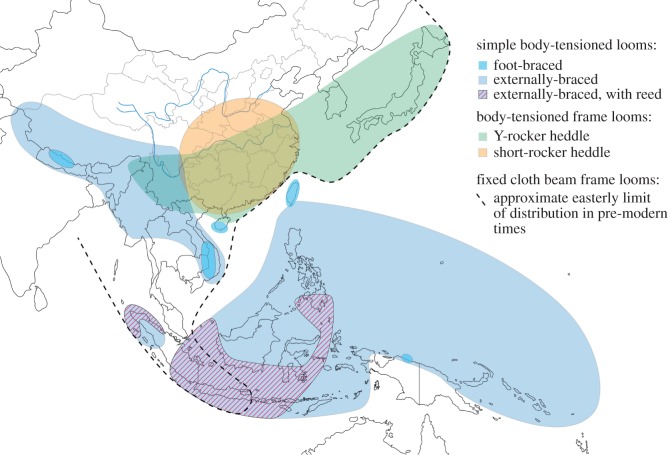
Geographical distribution of principal loom types (clades).

Lastly, we compared the complexity of the looms with data on societal complexity from the Ethnographic Atlas [33], to the extent that it is available for the communities we studied, and looked at the geographical locations of complex forms. Comparing loom complexity with societal complexity (figure 8) and geographical distribution (figure 9), it is apparent that complex looms with frames tend to be associated with larger and more stratified polities in lowland areas of the Asian mainland and the larger islands (Java and Sumatra). Conversely, communities in upland areas and smaller, more remote islands tend to have simpler, frameless looms. This relationship can also be seen in the plot of loom complexity (number of characters needed to describe the loom) versus societal complexity (number of levels of hierarchy). This association does not prove that there is a causal link between these two measures, but it does fit with our intuition about the characteristics of more complex societies.

**Figure 10.**
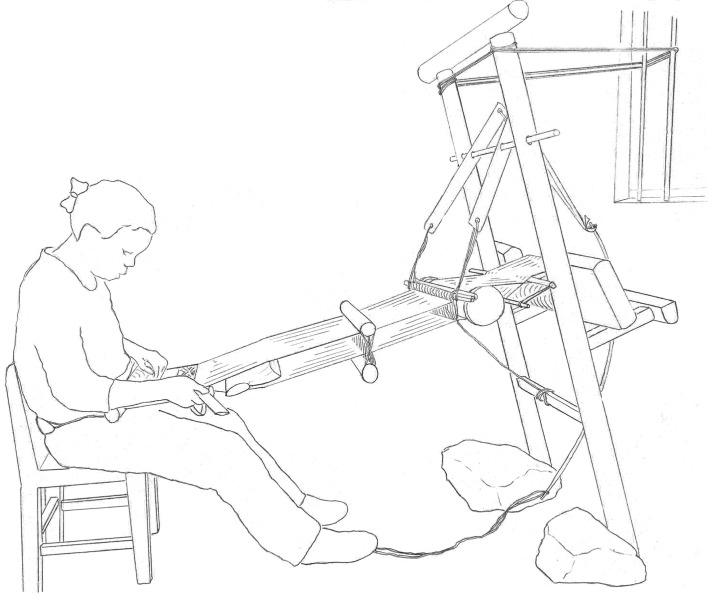
Drawing of the loom used by Miao weavers in the Panxian area of Guizhou province. This loom represents a simplification of the ancestral loom (at node F in the 70% consensus tree), via loss of the horizontal struts.

Remarkably, in three looms (the Miao loom in the Panxian area in China, the loom of Pa Then weavers in northern Vietnam and a frame loom used by Ainu weavers in Hokkaido), we found instances of present-day looms that appear to be descended from more complex precursors, in other words simplification has occurred. The clearest example of this is the loom used by Miao people from Panxian in China. This is a loom with a partial frame (figure 10), lacking the horizontal struts that are found in most looms in this lineage, that appears to be descended from an ancestral loom at node F (reconstructed in figure 6) that possessed a full frame with both vertical and horizontal struts. A similar partial loss of frame components has occurred in the Pa Then loom, and the same trend (loss of rear supports) can be seen in the Ainu version of this loom in Hokkaido. All of the groups using these looms live in remote, resource-poor regions and may have simplified earlier, more complex loom designs in order to use less timber (a scarce resource for all three groups) and/or to make the loom more compact or portable. The Miao in Panxian, for example, support the upright part of the frame by lashing it to a wall or tree with cords and substitute a chair (a multipurpose object) for the integral seat in a loom with a complete frame. This gives them a functional loom that uses less timber and that can be transported from place to place during seasonal transhumance or relocation, at the price of the loss of some rigidity and ease of use.

Despite the fact that a phylogenetic model can reproduce most of the data, some of the features of the looms in our set cannot be explained by pure vertical transmission. There are several instances where hybridization (horizontal transfer of technologies across lineages and between communities) seems to have occurred. In each case, the evidence for this is a group of looms in close geographical proximity that do not form a clade on the phylogenetic tree, but which nevertheless share some distinctive features. Two of these cases involve sharing of technologies between the cultures and looms that we studied, and the third involves a technology brought in from a different and apparently unrelated group of looms.

One example of hybridization was identified by us in a previous study [13], in which we showed that complex pattern-saving systems first arose in a Tai-Kadai loom lineage, and then spread across several geographically adjacent lineages, including the Han Chinese drawloom. These patterning systems (of which there are several variants) occur across several clades but (significantly) they are not present in the common ancestor of those clades. Given the complexity of these pattern-recording systems, independent invention is extremely unlikely, leaving horizontal transfer as the obvious answer, particularly as most of the looms concerned occur in the same region of SWC and Southeast Asia. These patterning systems consist of sticks (representing patterning wefts) embedded in a web of cords that are connected to the warp, such as the system on the Maonan loom in figure 2. They are time-consuming to make, but once made, they allow a weaver to reproduce designs precisely without having to refer to a template and pick out warps individually. This speeds up the weaving process and reduces the risk of mistakes. Though these systems appear to have been invented in a domestic context, they had obvious benefits for commercial workshops, which presumably provided the impetus for their adoption by Han Chinese silk weavers in a loom of otherwise different design (the silk drawloom, discussed in §7.4).

The second example of hybridization concerns the reed (a comb-like device used for separating and organizing warps). This feature seems to have arisen in the lineage of looms with raised frames and seats: it is present at node C, which is the ancestral loom for looms with complex frames. It is also found in a group of simple, ground-level looms, in central and western parts of Island Southeast Asia (Bali, Lombok, southern Sulawesi and other areas), but it is absent at the node at B6 for this clade and is therefore not ancestral in these looms. In this case the most likely explanation is that this feature has been ‘borrowed’ from more complex frame looms with reeds used in adjacent areas of Java and Sumatra, which trace their origins to the Asian mainland, and transferred to the ground-level looms. The driving force behind this transfer seems to have been the usefulness of the reed in weaving fine silk textiles with songket decoration (supplementary weft using gold thread), which were desirable status goods and important trade items in this region.

**Figure 11.**
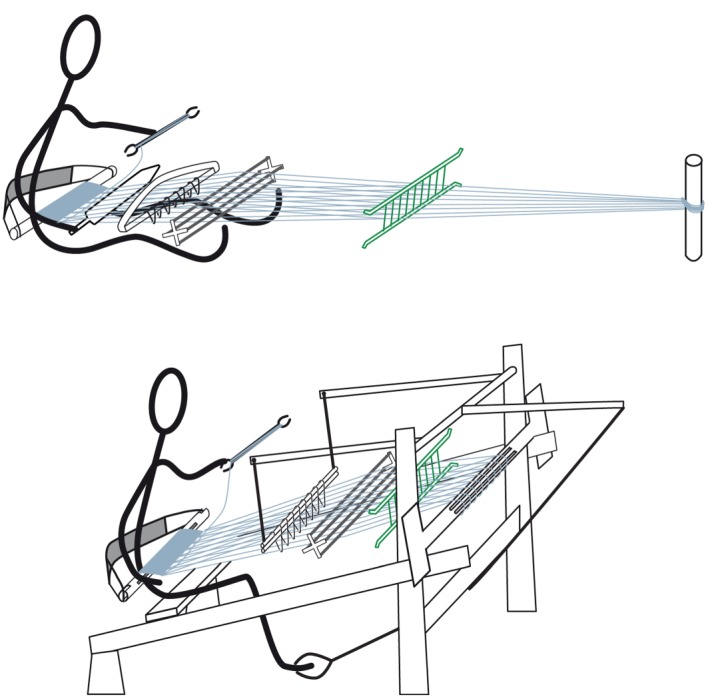
Ainu looms used on Hokkaido, with hybrid features. The loom at the top is a type that appears to be related to the looms of circumpolar weaving cultures and probably represents the simplest and oldest type of Ainu loom. The warp tied in a bunch to a stake, and a spacer plate (shown in green at the right) that spreads the warp out to the required width. This spacer plate appears to have been ‘borrowed’ from the older loom when the more advanced frame loom (below) entered Hokkaido. It replaces the reed (normally found directly in front of the weaver in looms of this type).

The third example concerns an unusual warp-spreader plate (resembling a reed, but positioned differently in the warp) that is found in an Ainu frame loom that traces its ancestry to node F (figure 11). Similar warp spacers are absent elsewhere in this clade, but are found in simpler, ground-level Ainu looms of a completely different type [34], which are not related to the looms in our study group. In these looms, the warp is tied to a point on single stick embedded in the ground, so a spreader is required to distribute the warps in a flat plane. This feature seems to have been ‘borrowed’ from this older type of loom. The reason for this borrowing does not seem to be driven by functional considerations: the frame loom does not (in principle) need a spreader plate near to the warp beam, since the warp beam itself does this job. The most likely explanation is that the Ainu, on adopting the more sophisticated frame loom, continued to use the techniques and components (fibre preparation and warp set-up) that they were already familiar with, including the spacer plate.

These hybridization events are represented by dotted lines between lineages in figure 5.

## 7. Discussion

Many discussions on the evolution of technology have centred on questions of how invention (the generation of novel technologies) and innovation (the spread of technologies) occur. Our analysis of the transmission processes of traditional weaving cultures, however, show that actual behaviours are mostly concerned with reducing errors and discouraging innovation. There are some precedents for these findings. Andersson [35] made similar inferences in his study of the transmission of Palaeolithic tool technologies, as did Palmer [36] considering the present-day culture of the Amish. Lewis and Laland [37] concluded that transmission fidelity is expected to be of critical importance based on theoretical considerations. A recent study by Perreault [38] demonstrates that the rate of cultural evolution is intrinsically faster than that of biological evolution, which suggests to us that the intrinsic error rate may also be greater. Considering these various findings together with our own observations, we conclude that the preservation of complex information and its faithful transmission are limiting factors in the development of complex traditions and technologies in pre-literate cultures. Furthermore, we suggest that error detection and correction will be found to be defining features of all complex, long-lived cultural traditions. These aspects have become unfamiliar in ‘modern’, literate cultures, since we take for granted that information can be easily preserved and duplicated as text or images, but for much of human history these tools were not available and the loss or degradation of important information was therefore the overriding concern.

There are some interesting parallels between our findings and theoretical conclusions stemming from information theory, particularly Shannon's analysis of signal transmission [39]. This theory addresses the general question of how a signal can be transmitted faithfully across a noisy channel. Cultural transmission is an instance of this type of process: the information that makes up the tradition is the ‘signal’ and day-to-day interpersonal interactions constitute the ‘noisy channel’. Shannon showed that error-free transmission is possible over an arbitrarily noisy channel via the use of error correction protocols. These rely upon redundancy, of which there are two types: repetition, and the encoding of information in larger blocks than are strictly necessary to encode the signal, which facilitates the detection of errors. Analogues of these techniques are found in the apprenticeship that novice weavers must serve. The novice ‘over-learns’ her craft by repetition over a long period. An older weaver engages in very little active ‘teaching’, but devotes her energies to correcting mistakes. Weaving processes are organized into concatenated ‘blocks’ of procedures, which include ritual content and ‘redundant’ information that does not appear to influence the outcome, but which must nevertheless be copied precisely. We suspect that errors are easier to spot and to correct in highly codified/ritualized procedures, versus more informal instructions. This is because an informal or imprecise set of instructions has multiple potentially correct versions, whereas a highly codified set has only one, making it easier to check. For example, in traditional weaving cultures, there is considered to be only one ‘right’ way to warp a loom, even though there are other methods that might achieve an apparently identical result. The steps must be carried out in a certain order, and the procedure often includes ritual observances such as offerings to village gods, as well as the observance of taboos.

Some of the issues related to the transmission of cultural information were discussed by Pocklington and Best [40, p. 81], who argued that the ‘units of selection’ of culture are ‘the largest units of socially transmitted information that reliably and repeatedly withstand transmission’ and that these must be ascertained empirically rather than deduced *a priori*, a conclusion that our work supports.

### 7.1. Is ‘bias’ a factor in the transmission of weaving cultures?

A widely used class of quantitative models of cultural transmission introduced by Cavalli-Sforza and Feldman [41], and Boyd and Richerson [42], include the concept of ‘bias’ as a central feature. Bias means choices made by individual members of a population concerning who and what to copy. In principle, it is a defining characteristic that can distinguish cultural evolution from ‘biological’ evolution of organisms, since the latter involves no choices and is unbiased by definition. Models of cultural evolution that include bias have become very influential in the study of the transmission and evolution of human culture (see Mesoudi [43] for a recent review). Several different kinds of bias have been proposed, including content biases (selective transmission based on the perceived benefits of different practices) and context biases (selective transmission based on assessment of the transmitter, such as prestige bias and conformist bias).

As outlined above, in our study of transmission processes in weaving communities, we find little evidence that apprentice weavers are able to make choices of this kind, particularly where technique is concerned. Instead, the transmission process is geared to reproducing the tradition (consisting of information embodied as procedures and as artefacts) whole and intact, with a minimum number of errors or modifications. As noted, the main mode of transmission is vertical and is said to be ‘unbiased’ (despite the fact that the novice weaver can be said to have a strong bias towards conforming to the cultural norm). Who learns, what they learn and how they learn are determined by the tradition itself, and are not a matter for individual choice.

This is not to say that bias is never a factor in the transmission of weaving. The instances of horizontal transfer of weaving technology between lineages that we uncovered, though rare, can only be accounted for by the operation of content bias (recognizing and adopting a superior technology). The transmission of information between households (within a community) may also be subject to prestige or conformist bias. Though we did not find direct evidence of the operation of bias during our fieldwork, there is indirect evidence in the rapidity with which many weaving communities adopted commercial yarns and dyes (particularly for daily-use textiles) during the twentieth century. Changing fashions within communities and the partial adoption of western-style clothing are probably also evidence of biased horizontal transfer within the community.

The degree to which ‘content bias’ operates, and hence the degree to which innovations from outside the community are adopted, seems to depend on the type of information and its place and importance within the weaving tradition. As noted, weavers are reluctant to make changes in looms or technique, which seems to be linked to the complexity of this information and the difficulty of transmitting it intact. Errors are costly, and the rewards of invention are uncertain. By contrast, new types of commercial yarn and dyes, particularly for daily use clothing, can be adopted relatively easily, since they tend to simplify the weaving process. Their adoption may also be linked to a different mode of transmission (peer-to-peer) and to competitive pressures within the community, particularly between young, unmarried women, since clothing is highly visible and communicates status.

Despite this conservatism, our analysis of loom technologies shows that major changes have occurred in some lineages, considered over a long time period. Our observations on patterning systems (and the scarcity of individuals who can make or modify these) suggest that weavers who are talented enough to modify and improve upon loom technology are very rare indeed, and it is perhaps not surprising that we did not meet such people in our research. They must exist (or have existed), however, to account for the accumulation of novel features in present-day looms.

### 7.2. Transmission within versus between communities

Our study highlights an issue that has been discussed by Mace and Jordan [44], relating to the definitions of ‘horizontal’ and ‘vertical’ transmission. The term ‘horizontal transmission’ must be divided into two types with very different outcomes as regards cultural evolution: intra-community and extra-community transmission. The former, which occurs via intermarriage and via social exchange within communities, is frequent and (as noted) is essential to the formation of a homogeneous cultural ‘taxon’. Its role is analogous to that of horizontal gene transfer mechanisms (such as sexual reproduction) in species formation in biology. This process must be distinguished from extra-community transfer, which tends to blur the differences between communities, and which is suppressed by cultural barriers and taboos, particularly barriers to intermarriage between communities.

Our macro-level study reveals the consequences of micro-level transmission processes. Loom designs and operating skills are transmitted with a high degree of fidelity from generation to generation, resulting in distinct technological lineages. However, in spite of the barriers to modifying technologies, and the barriers to cultural exchange between communities, our results also show that inventions and the transfer of technical innovations across community boundaries must occasionally occur. The likelihood of observing these rare events ‘in the field’ is low, however: for example we estimate that a cross-cultural transfer rate of less than once-per-generation would be sufficient to reproduce the results that we see.

### 7.3. The evolution of technology

The phylogeny of looms that we have deduced here does not support the notion that technological change has an inherent ‘direction’. Technological progress (increased complexity) is not inevitable. Some lineages in our study grew more complex, others stayed almost the same and some (in a few cases) become less complex over time. There appear to be more varied and complex looms today than was the case at the earliest period, but this is best explained as a combination of the starting point (the earliest loom was relatively simple) and external factors (the emergence of complex polities that were able to support complex, resource-intensive technologies). We expect that this ‘lack of inherent direction’ will be found to be generally true in respect of pre-modern technologies and craft traditions. Furthermore, we would expect long periods of technological stasis to be the norm in the absence of external factors (such as competition within or between cultures) that might drive increased technological complexity.

### 7.4. Domestic versus workshop production

**Figure 12.**
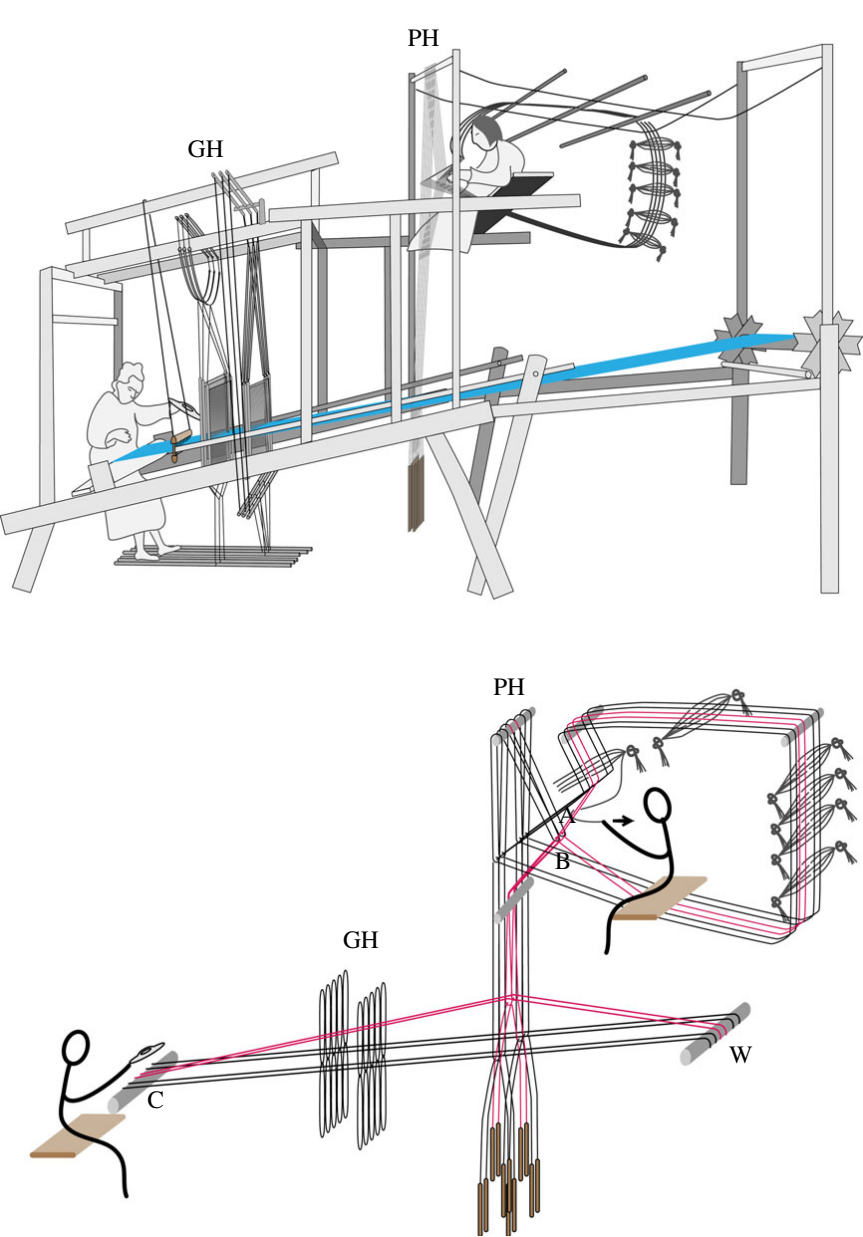
Drawing of the greater drawloom (above) and schematic diagram of the connections of the patterning system to the warp (below). The weaver (on the left) inserts the weft and operates the ground-weave heddles (GH). An assistant (on the right) operates the pattern heddles (PH). This system records warp lifts using bunches of cords embedded in a circular pattern system. The assistant uses each cord in turn, pulling it towards herself to raise each set of warps via the linkages shown. As she does so, she removes the cord from the opening A and reinserts it at B, preserving the pattern for future use. A small number of pattern leashes and warps are shown in the schematic diagram: drawlooms typically hold hundreds of leashes and record thousands of individual operations. C, cloth beam; W, warp beam.

Though most of the looms in our study were used domestically, three were used in commercial workshops: these are the stepping stone loom from SWC, and the two types of drawloom that were used in silk-weaving workshops in several parts of China, mainly producing luxurious, patterned silks for use by the imperial court. These looms are among the most complex in our study, the two drawlooms incorporating patterning systems capable of recording thousands of individual warp lifts, corresponding to rows of pattern wefts in the finished textiles (figure 12). Their exact chronology is not precisely known, but their development seems to have taken place relatively rapidly compared with other looms in our study. While we have no direct evidence for why this occurred, circumstantial evidence suggests that the pressures of commercial production accelerated the development of these looms.

### 7.5. The role of human agency

A reviewer of an early version of this paper asked us what role human agency plays in technological change, and whether our work challenges traditional views that technological change is owing to the creativity of individuals. This concern is sometimes raised with regard to phylogenetic studies of change. In fact, human invention plays an essential role in our model. Change comes about as a result of invention (inventing a new technology) or innovation (adopting a new idea from an external source). Both of these processes are examples of human agency. The fact that the inventors and innovators in traditional crafts are (in most cases) nameless and unrecorded makes them no less important.

This topic is an example of a broader question that concerns ‘explanations’ for complex phenomena. For example, we can ascribe a given technological improvement in a loom to the genius of an individual weaver or group of weavers. We could also point out that the most complex looms in our study arose in commercial settings in larger polities and that commercial and economic factors probably played a role in providing the conditions under which inventions could occur and take root. These explanations are not incompatible; rather they operate at different scales (micro and macro, respectively). Explanations spanning multiple scales are routinely employed to account for complex phenomena, for example, the history of the Industrial Revolution in the United Kingdom has been written about in terms of the biographies and contributions of individual inventors, and in terms of the economic and social factors that made it possible. These are complementary and equally valid ways of examining the question.

### 7.6. Diversity in technological development

A distinct difference between our approach, and more conventional ‘historical’ narratives of cultural and technological change, is that we have explicitly considered the range and diversity of technological solutions that have arisen to a given problem, and as a result we have been able to show how technological change proceeds on many different pathways simultaneously. This is in contrast to conventional historical-narrative treatments that tend to emphasize a single pathway, or one or two competing pathways, usually leading to the triumph of one particular technology. For example, accounts of the development of weaving in China, of which a recent study edited by Kuhn [45], is an important example, mainly focus on one type of textile (luxurious silks produced to imperial commission and employed in international trade) and one technology (the drawloom) that is widely (and with some justification) regarded as the crowning technological achievement of this tradition. Our analysis presents an alternative view, revealing diverse traditions and technologies operating within the borders of China and East Asia as a whole, and the connections between them.

## 8. Conclusions

Our conclusions are as follows.

1. We have shown that traditional weaving cultures in Asia, of which loom technology forms a central part, are self-propagating systems with internally defined transmission mechanisms incorporating error correction. These features have enabled their persistence over millennia. We believe that these characteristics will be found to be quite general in the study of long-lived and complex traditions in human cultures.
2. Complex craft traditions, of which weaving is an example, consist of more than just the skills and knowledge of their participants and include embodied information (tools, templates and domesticates).
3. The main mode of transmission of traditional weaving technology is vertical (mother to daughter) and is unbiased. Despite this, biased transmission occasionally occurs when innovations travel between communities and plays a role in other aspects such as clothing design and decoration.
4. The particular group of technologies that we studied display branching evolution. Some lineages cannot be completely explained, however, without invoking hybridization (sharing of technologies between lineages, resulting in a reticulated pattern of descent). These events are rare (and consequently difficult to observe in practice) but can result in important and long-lasting changes in lineages.
5. Some lineages developed highly complex looms; others showed lesser changes or virtually no change over millennia (stasis). A few showed losses (simplification), confirming that technological change has no inherent ‘direction’. Today, a diversity of simple and complex forms exists, a pattern that has a general resemblance to that produced by biological evolution.
6. All other things being equal, more complex societies seem to be associated with the emergence of more complex weaving technologies.

It is important to note that our study focuses on one of the most durable aspects of weaving culture (loom technology). Had we instead chosen to investigate more ephemeral aspects (such as clothing fashions), in which peer-to-peer transmission plays a bigger role, we might have found quite different patterns on a macro scale. We believe our conclusions will be found to be broadly applicable, however, to the cumulative aspects of human culture, which include humanity's most impressive achievements.

## Supplementary Material

S1 Background, methods and data supplement

## Supplementary Material

S2 Looms data sources

## Supplementary Material

S3 Looms characters and short codes

## Supplementary Material

S4 Looms data file

## Supplementary Material

S5 Looms ancestral states

## Data accessibility

All the data used in our study are included in the electronic supplementary material.
