# Supplementary material for: The evolution of an ancient technology
Source: R Soc Open Sci. 2017 May 31;4(5):170208. doi: 10.1098/rsos.170208 (PMC5451833; doi:10.1098/rsos.170208)
Supplement: S1 Background, methods and data supplement [file rsos170208supp1.docx]

**The Evolution of an Ancient Technology by Christopher D Buckley and Eric Boudot: background, methods and data supplement**

**1. A primer on looms and weaving**

Weaving consists of the interlacing of flexible materials to make a coherent two-dimensional structure. The interlacing of yarns, which lack natural rigidity, is most conveniently done on a frame of some kind, called a loom. The frame tensions one yarn (the warp) between two supports, with a second yarn (the weft) interlaced with it at a right angle. The simplest weave is called plainweave, and consists of a strict alternation (over, under) of warp and weft in both dimensions. To make this weave, the warp yarns are divided into two sets that are raised and lowered alternately.

All of the looms that we consider in our study use the same basic system, or variants of it, for accomplishing this interlace (Supplementary Figure 1). The warp is horizontal (or at a shallow angle to the horizontal), stretched between two parallel beams (called the warp beam and the cloth beam). A rod (shed stick) is placed in the warp and defines one opening (natural shed) through which the weft is passed. The other opening is created using a device called a heddle, which allows one set of warps to be pulled through the other set to create a reversed opening (countershed) through which weft is passed. Using these two openings alternately for weft insertion creates plainweave. In more complex looms the shed stick may be replaced by a second heddle, which pulls in the opposite direction to the first.


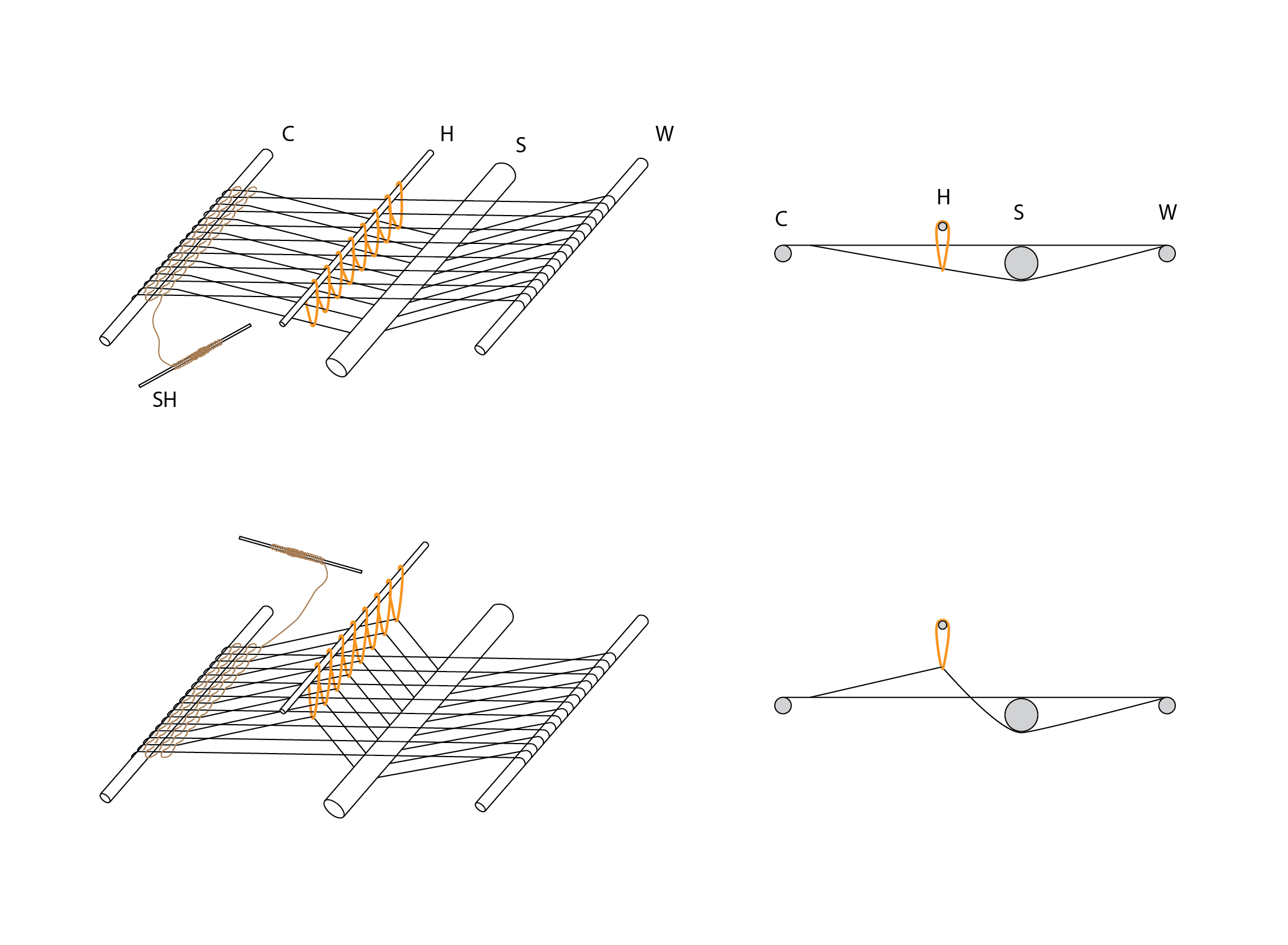


Supplementary Figure 1. Basic weaving processes

There are several ways in which warp yarns can be mounted between two beams (Supplementary Figure 2), of which two methods are found in the group of looms that we chose to study. One method is to make a continuous loop by winding it between the beams (circular warp). This is gradually shifted around as weaving progresses, making a tubular piece of cloth. The other way is to wind the warp onto the warp beam, gradually unrolling it as weaving progresses and winding the woven cloth onto the cloth beam (flat warp). This system is more time-consuming to set up, but can be used to weave a much longer piece of cloth.


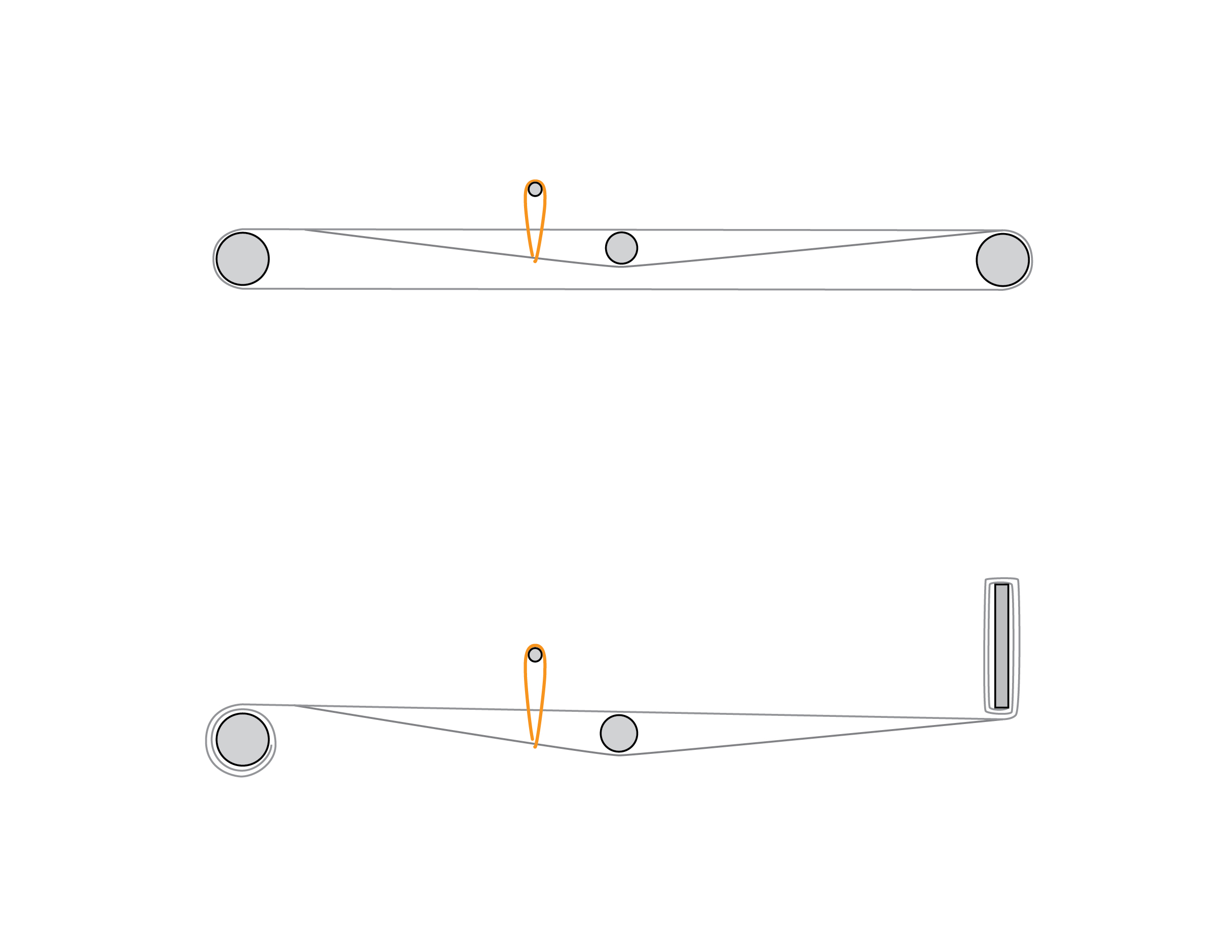


Supplementary Figure 2. **Loom** **warping types.** Above: circular warp. Below: flat warp, wound onto the warp beam at the right.

This basic system is subject to many variations. Additional sticks and heddles may be added for lifting subsets of the warp, in order to create patterning. The system may be embedded in frames of various kinds. Mechanical aids can be added to allow the weaver to operate the heddles with her feet rather than her hands.

Other types of loom are occasionally found in our region that use different methods for opening the warp and making the interlace, and that are used for different purposes:

1. Looms with rigid heddles and/or spacer-plates

2. Card-weaving looms

3. Vertical carpet and rug weaving looms

4. Horizontal looms attached to four stakes in the ground

The first two types are used mainly for weaving narrow strips of cloth such as belts and animal trappings. They are found sporadically across Europe, Central Asia and Asia. The third and fourth types are mainly found in the far west of our region. They seem to be incursions from Central Asia, where they are the main types of loom for weaving cloth.

The wide variation in loom types in different regions of the world implies that weaving technologies are not ‘obvious’, since if they were we would expect to see similar solutions occurring everywhere.

**2. Archaeological remains of looms in East Asia**

The oldest remains of weaving equipment in Asia that have been discovered to date are a group of wooden tools found at Hemudu, a Neolithic site in Zhejiang province in China, the oldest layer of which has been dated to around BCE 5000. Some authors have reconstructed these items as a body-tensioned loom, but due to their incomplete state, and it is not possible (in our opinion) to reconstruct exactly what this looked like.

The oldest remains of a loom that can be reconstructed with a reasonable degree of certainty are a set of loom parts made of jade (Supplementary Figure 3) that were found in a tomb of the Liangzhu culture, also in Zhejiang province, that date from around BCE 2500 [13]. These parts were found in two groups, separated by around 30cm, in an orientation that suggests that they were finials for a group of wooden weaving implements (now decayed), all of which had a width of around 30cm. These have been interpreted, correctly in our view, as remains of a back-tensioned loom of the foot-braced type. The short warp beam with a flattened oval profile implied by the remains would have been well suited to bracing against the weaver’s feet, but not well-adapted for bracing against external supports or fixing within a frame.


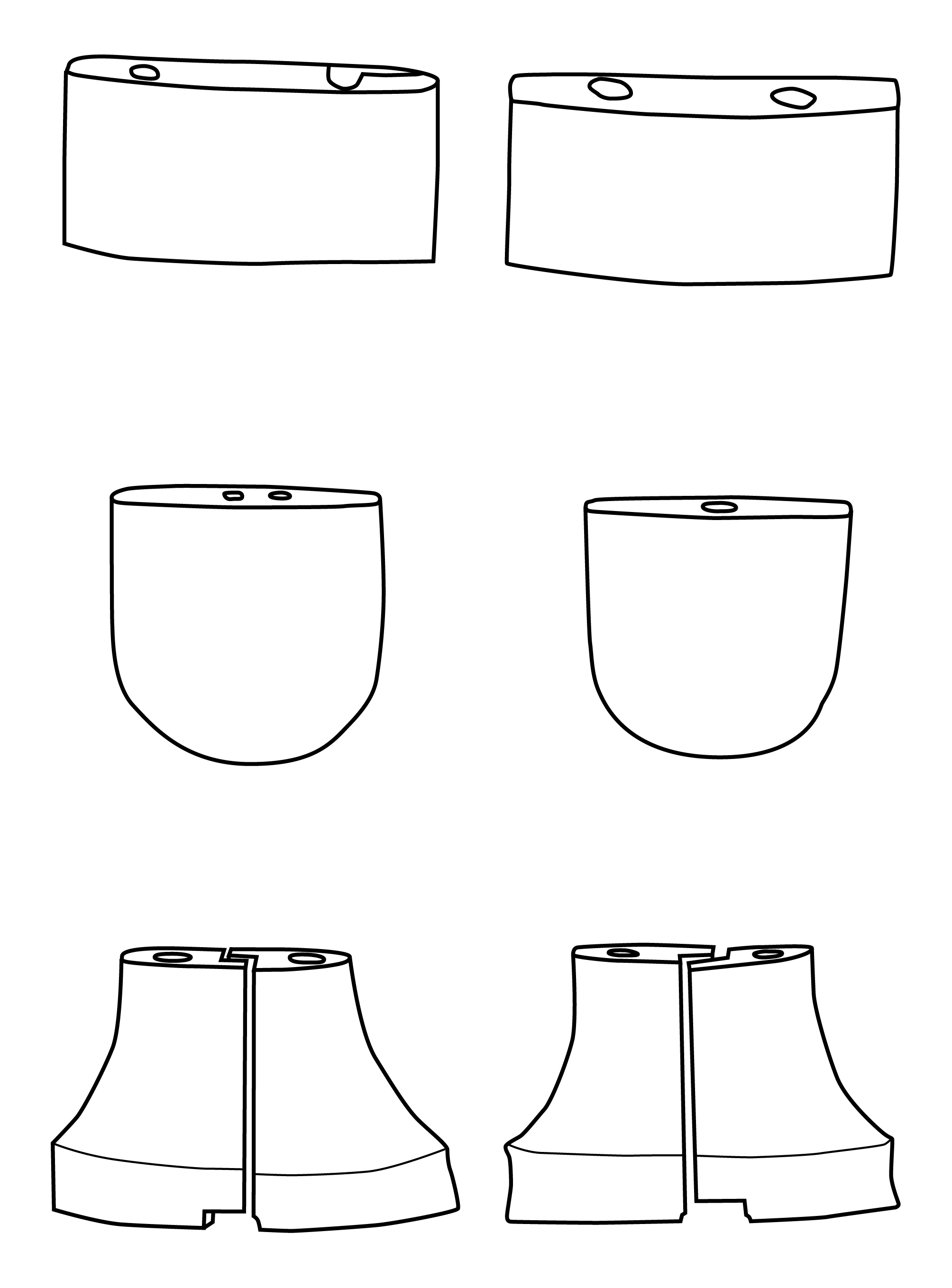


Supplementary Figure 3. **Drawings of loom parts from a tomb of the Liangzhu culture** (BCE 2500 approx.). The components appear to be finials for parts of a body-tensioned loom. From the top: warp beam, sword beater, cloth beam (split into two parts in order to clamp the cloth). After Boudot and Buckley [13].

Remains of similar foot-braced back-tensioned loom parts have been found in tombs from the Dian kingdom (BCE 400 to 100 CE) in what is now Yunnan province in southwest China, described by Vollmer [1979]. The Liangzhu and Dian remains of this type of loom suggest that it formerly had a much wider range than it does today. The occurrence of this loom in several archaeological contexts, together with the lack of remains of any other kind from the earliest periods and the relative simplicity of the design led us to use this loom to identify the root of our Bayesian phylogenetic network (see Materials and methods section).

More complex looms with frames are attested in the archaeological record from the Han dynasty onwards:

A group of wooden loom parts found in the Luobowan M1 tomb in Guangxi province, China [Feng Guangyu 2006, 104-106], dated to the Western Han dynasty (BCE 206 – 25 CE), belong to the group of looms with a common ancestor at node F in the Bayesian tree. The defining features of this group of looms include a large warp beam with paddle-shaped ends, and a single treadle. This loom is also depicted in a carved stone panel from a tomb from Chengdu, in Sichuan province, China, of approximately the same period.

Another kind of frame loom, that has two treadles and different mechanical linkages, is attested by several Han dynasty tomb carvings in Shandong and Jiangsu provinces, and appears to have been a common and widespread domestic loom during the Han dynasty [13]. These looms appear to belong to the clade defined by node E in the Bayesian consensus tree.

Recently, the remains of looms with large, box-like frames, dating to the Western Han dynasty, have been found at Tumen, near to Chengdu. These looms appear to be an early type belonging to the clade defined by node D.

From the remains of these looms, which include a wide variety of sophisticated designs, we conclude that a major radiation of loom designs based around a raised frame with a seat (node C and subsequent looms) took place on the Asian mainland prior to the Han dynasty, ie more than 2200 years ago.

**3. Cultural transmission of weaving traditions: review of published sources**

Ethnographic studies on individual communities [26-29] provide further perspective on the transmission of weaving cultures, in addition to the two area studies mentioned in the main text. Though the authors of these studies did not explicitly set out to address ‘transmission’, they placed themselves in the position of novice weavers, and experienced the learning process at first hand.

3.1 Rupshu people, Ladakh Himalaya

This region and its people, studied by Monisha Ahmed [26], are unique in that both men and women weave (male weaving traditions are uncommon in Asia, particularly in a domestic context), but do so using quite different looms and associated tools. The female loom is a ground level body-tensioned loom that is similar to many other looms in our study area. The male loom is a variant of a Central Asian loom that is tensioned by staking it out on the ground with four pegs. Men weave heavy-weight fabric made from goat or yak hair, used for making tents and bags, while women weave lighter weight textiles from wool, used for clothing and sleeping mats. The female loom is included in our study, but not the male loom since it belongs to a different and apparently unrelated group of looms.

The Learning Process

‘From a young age girls in Rupshu start helping their mothers spin and clean wool, and around the age of fifteen they begin learning to weave. This knowledge is transmitted to them by their mothers, grandmothers, or aunts. Men learn to weave at a later age than women, generally around the age of twenty or even as late as thirty. In much the same way as women, they are taught by their elders.’ [26: p77].

Ahmad gives a detailed description of the process of learning that one girl, undergoes. She is taught to set up the loom by her mother and grandmother, who assist her in setting up the loom for the first time. She learns weaving as a ‘hands-on’ task, one that she has observed many times but not tried for herself before. Learning to weave is not a matter of choice, it is an expectation within the community: ‘At first a young girl finds it tiresome to weave, but gradually she learns the importance of her task, which is to weave clothes for the entire family and later for her new home when she gets married. Once the girl has become competent at her weaving, it is common for a mother to leave the entire family’s weaving to her young daughter, who generally works under her supervision.

Girls begin by learning to weave nambu, the plain woollen cloth used for all clothing. In the early stages they are given coarse wool to weave with, using finer wool later on as their skills improve. In Rupshupa culture it is expected that women will weave: the onus on men is less strong. There are numerous traditional injunctions that explain why it is essential for a woman to weave, and the dire consequences of not weaving: ‘There is always the danger that women who don’t weave will become the demoness (blud-mo) again … that is why we women must keep weaving’ [26: p93].

Rupshupa culture views men and women’s work as complementary and life as a cooperative endeavour [26: p76], but there is also a strand of thinking that views women as dangerous, and regards weaving as a means for their control: ‘It is said that a woman who is preoccupied and absorbed with her weaving will have little time to think wicked thoughts or commit sinful actions’ [26: p93]. No such constraints and injunctions exist in relation to men’s weaving.

Numerous songs and legends attach to weaving, and the Rupshupa seem fond of analogies (sexual, spiritual and so on) that explain the place and importance of weaving. Weaving is also used as a metaphor to illuminate aspects of life: a family group is regarded in the light of a piece of woven cloth: ‘The woven cloth also defines one’s lineage, where the warp is perceived as being male and the weft female.’ [26: p99]. This metaphor has a literal counterpart in the designs of cloth woven by men, the sequences of stripes (yud) belonging to individual families and in some cases to individual weavers. A yud is regarded as belonging to a familial tent, and passes from father to son along with the tent. When a son moves away he modifies his yud, though it remains similar to the one he wove in his familial tent [26: p154].

Weaving skills are regarded as a pre-requisite for marriage: ‘The weaving of cloth is a measure of a woman’s worth in Rupshu, and a woman who does not weave, or one who does not weave well, is not considered a good bride.’ [15: p98]. Ahmed recounts the story of a failed marriage, lasting only two months, in which the bride’s weaving skills were blamed, along with her general lack of industriousness, for the failure of the marriage. (26: p98). Dowry items consist largely of woven items, woven by the bride’s mother and father or by the bride herself, supplemented by gifts from other relatives. An exact record of these goods is kept during marriage and other ceremonies, detailing the reciprocal obligations of the families.

The Loom

The loom used by female weavers is a ground-level, body-tensioned loom, lacking a frame. The only major change to the loom design in recent years is the addition of a metal edge to the beater, which increases its effectiveness and durability. Ahmed notes that looms are inherited within families, and that:

‘one loom can be used for generations. Men make looms for both men and women using wood, rope, wool and metal. The Rupshupa say there is little difference between the structure of the looms that they use now and those used in the past, the only change is that the new looms have more metallic parts as these are now easily available in the shops in Leh.’ [26: p83].

Transmission of motifs

The designs woven by Rupshupa weavers are relatively simple, consisting of stripes and some simple decorative motifs on looped-pile rugs. The precise sequence of stripes on bags and other items is unique to each household, though all the designs used within the community are broadly similar in type and layout.

3.2 Lamalera, East Nusa Tenggara, Indonesia

Ruth Barnes [27,28] studied the weaving traditions of village of Lamalera on the island of Lembata. Weaving is of particular importance in this village since the steep rocky coast is unsuitable for agriculture, so the village relies on fishing and textile production, the products of which are traded for agricultural produce with nearby communities. Nearly all women in the village weave, using a traditional back-tensioned loom at ground level, of very simple design. Cloth is decorated using the ikat technique, applied to the warp before weaving.

Barnes notes that weaving is the exclusive realm of women: ‘In the Lamaholot region it is definitely and exclusively women who manipulate costume: they weave it, they know which patterns are appropriate, they sell it, they offer it as a ceremonial gift which is part of every marriage, and they receive and judge it at such a ceremony of bridewealth exchange.’ [27: p6].

The Learning Process

The process of learning to weave is lengthy, and begins at an early age with activities that are indistinguishable from play: ‘Dyeing would be the first activity relating to cloth production which a little girl joined in; I was told several times how much fun it is for a small child to meddle with the dyes and get her hands all covered in red or black.’ [27: p32].

‘Little girls will start to spin cotton at the age of four or five. They can go on to weaving when they are sixteen or seventeen. The last thing they will learn is the ikat technique.’ [27: p48]. ‘Virtually all young women will have worked on some traditional cloth by the time they marry, either helping with the ikat, they dyeing or the weaving. They will have done so assisting their mothers or aunts, and will not have had an initial role in the production.’

Although weaving activities tend to take place communally, the main teacher in most cases is the mother or another close relative: ‘Women may actually be reluctant to apprentice young girls in their art if they do not belong to their household’ [27: p32].

Even after marriage, a woman is likely to return to her mother (rather than her mother-in-law) for advice and guidance on weaving questions.

Most learning takes place by the novice weaver observing and copying what her teacher(s) do, rather than via verbal instruction. The way in which the loom is used is identical (or nearly so) for all the weavers and there is no secret about how it is done, but dye recipes and procedures are accompanied by a certain amount of secrecy: ‘Although the ingredients which go into the preparation of dyes are common knowledge, the actual procedure is something which is passed down from mother to daughter, to be learned from experience rather than something openly discussed.’ [27: p32]. There is an element of competition between weavers, particularly in the production of the most prestigious bridewealth (adat) cloths, and colour, particularly the depth of the red colour from Morinda dyeing, is an important criterion for judging the quality of a cloth: ‘The adat cloth is supposed to be strictly traditional; only the patterns which have ‘always’ been used locally are permitted to appear in the ikat decoration. No external influence is now allowed to show, even when this would be the traditional design among a neighbouring group of people’. In contrast a ‘festive sarong’ may show ‘any design which catches the weaver’s fancy’ [27: p69].

The weavers are stern critics of the quality of finished textiles: ‘… they are fully aware of differences in craftsmanship between similar pieces, and they do not like carelessly done ikat. This became quite clear to me one day when I was looking in passing at a cloth hung out to air, which had a design I had known by name but had never actually seen before. The woman accompanying me explained the pattern, but then said in a rather abrupt way: “Now let’s go on. This ikat is terribly done and I can’t stand to look at it any more”’. [27: p43].

Transmission of motifs and layout

When tying ikat, weavers relied on memory, or copied old cloths.

The Loom

The loom used for weaving is relatively simple, as noted. Most of the parts consist of bamboo or wooden rods that can be easily substituted or replaced. The weaver’s sword-beater is the main exception to this, since this is a critical tool (used for opening sheds in the warp and beating-in of wefts). It is made of durable hardwood such as tamarind: ‘A sword is passed from mother to daughter and is quite likely to survive other parts of the loom, which can be replaced quite quickly, with the exception of the cloth beam, which needs a certain amount of carving. A sword is usually made for a woman by a close male relative; it is always a gift rather than part of a barter exchange. The different parts of a loom are rarely made by one person only.’ [27: p37].

Spread of patterns via marriage alliances

Barnes notes that some patterns are regarded as the common property of the village, while some are said to be clan-specific (they are only supposed to be woven by members of certain families), but she found that the reality did not reflect the oral tradition: ‘patterns, although they *belong* to a certain clan, can spread throughout the village. At least in Lamalera, the ikat patterns seen on the women’s sarongs do not divide the community into defined groups.’ [16: p75]. ‘there exists a definite prohibition on using patterns inconsistent with a woman’s clan affiliation … This apparently rational, clearly defined position is in complete contrast with what the observer finds in reality’ [28].

3.3 Batak weavers, Sumatra, Indonesia

Sandra Niessen spent an extended period studying the weaving traditions of several Batak groups living near Lake Toba in northern Sumatra, detailing her findings in her monograph on Batak weaving. Niessen ‘apprenticed’ herself to an experienced older weaver, and thereby experienced the learning process at first hand. Tellingly, at the outset of their relationship her teacher insisted on ‘adopting’ Niessen and refers to her as her ‘daughter’ during the teaching process [29: p541]. Niessen also observed other young women learning to weave, and the weaving life of the Batak communities in general.

Learning to Weave

Niessen notes that Batak society has clear expectations of its weavers: ‘When a Batak weaver sits at a loom, her goal is to make a cloth that meets social expectations’ [29: p28]. ‘When they learn to make textile design types, the weavers must learn not only the design characteristics to which their cloths must conform, but also their creative latitude. All this is laid down by convention: informal, but clear, social agreement that is generally known and unquestioned. In this regard, Batak textiles are, in a very important way, social and not individual creations.’ [29: p28].

The process of learning to weave begins at a young age with yarn preparation, the first task being to wind weft onto bobbins (prior to being used for weaving) [29: p430].

Much of Batak weaving involves processes that are characterized by ‘correct’ ways to do them, which must be learned by observing and doing. Older weavers are quick to correct ‘mistakes’. Niessen, who had some prior weaving experience before learning with the Batak, describes the reaction of her teacher when she inserted the weft from the ‘wrong side’ of the loom: ‘A Batak weaver lays in the weft from the right or from the left, depending on which shed (opening in the warp yarns) she has. [She] did not tell me which way to do this (because it was too obvious to her? because she taught by example and such lessons were not verbalized?), but when I threw in the weft from the ‘wrong’ direction she was uncompromising. She cut my weft yarn immediately and had me start again.’

Niessen reports how a fellow novice weaver described her experiences with learning the ‘correct’ way to repair a broken yarn: ‘[she] told me that her teachers in the village were very strict about this. They pinched her arm if she could not repair a broken strand properly’.

The relationship between the novice weaver and her teacher has a formal dimension in Batak society, and is accompanied by certain rituals. Niessen describes how the novice and her teacher are expected to cooperate in the tricky task of building a pattern heddle with two rods: ‘It was a difficult task demanding great precision. It also called for the ritual consumption of *nitak gurgur*. As the student, I was to supply these rice cakes to the maker of the supplementary weft, accompanied by the words *Hipas ho na mangulaon bonangna on* (‘Well-being to you who is working on this yarn’).’ [the teacher] had not warned me of this duty in advance, so I was a day late in supplying the rice cakes and by this time she had finished the pattern heddles. She graciously accepted them anyway, ate one, and rubbed a portion of it onto the ends of the pattern rod heddles.’

Niessen found no evidence of any secrecy between neighbours concerning weaving activities and methods, despite the fact that Batak weaving has significant commercial value to many of the weavers.

3.4 Studies from other regions

The studies we have reviewed above relate to the region of East Asia where our looms survey is located. Studies from further afield, such as that of weavers in Mexico by Greenfield and Lave (1982) and Greenfield (1999), and Tehrani and Collard’s study of Iranian tribal weavers [9], suggest that these findings are not limited to the East Asia region. Greenfield found that transmission amongst Zincatan weavers was mainly from mother to daughter, and noted that:

‘the learning process was a relatively error-free one, in which the teacher, usually the mother, sensitively provided help, models for observation, and verbal direction in accord with the developmental level of the learner’

she also noted: ‘the apprenticeship process was highly structured by the older generation and did not allow room for learner experimentation and discovery, the method of informal education (or apprenticeship) was well adapted for the continuation of tradition, the maintenance of the status quo.’

Tehrani and Collard [9] investigated weaving traditions amongst nomadic tribal groups in Iran, and found that techniques are also passed mainly from mother to daughter. They found that motifs may be shared amongst family members and peers (oblique, within-group transmission), but exchange of techniques and motifs between groups is limited or absent because of a strong preference for marrying within the group (endogamy). Significantly, they also found that even though the means for the transmission of motifs between groups exists (via commercial carpet weaving workshops employing weavers from several tribal groups), the textiles they examined showed little evidence of copying between tribal groups, implying the existence of other barriers to cultural exchange.

**4. Materials and methods: additional notes**

We examined horizontal looms from 85 present-day weaving traditions, chosen to represent the widest possible variety of loom designs that we could find, over an area including Southeast Asia, East Asia, Island Southeast Asia and parts of Oceania. We also included in our survey the archaeological remains of the oldest loom that can be positively identified as a back-tensioned loom (discussed above) from the Liangzhu culture. Other archaeological remains of looms provide useful markers for the time-depths of key technologies, but were not included in our analysis.

Loom diversity is not evenly distributed geographically, so some geographic regions are over-represented because they have a greater diversity of loom types. Data sources for each loom are listed in the Supplementary Material S2.

Each loom was analysed in terms of its physical components, their interconnections and functions. Since our emphasis is on function, aspects such as materials used to make the loom (wood, bamboo etc) and decorative features were not included. A master list of around 400 distinct features (characters) was identified, and each feature was marked as present (1) or absent (0) for every loom.

The matrix of data (Supplementary Material S4), in the form of a Nexus file, was subjected to a Bayesian phylogenetic analysis. This consists of a search to find the best-fit tree model for the data. This was carried out using the MrBayes software [31], employing a Markov-Chain Monte-Carlo (MCMC) method, which samples trees and selects them according to the posterior probability that they could have generated the observed dataset. For this search we used ‘flat’ prior assumptions, and a search using 20 million chain steps, of which the first 5 million were discarded (‘burn-in’). Character changes were modelled using a gamma function with 6 steps (the maximum allowed by the software). This allows considerable variation in the rate of change of characters. We chose this method because of our expectation that some loom features (such as the frame and warp support method) would prove to have been stable over long periods, whereas others (such as some patterning arrangements) would be found to have changed relatively fast.

The output from the Bayesian analysis was summarized using the Mesquite software (Maddison and Maddison 2011) in the form of a consensus tree that includes splits present in 70% or more of the output trees (70% consensus tree). Mesquite was also used to calculate Retention Index for this network.

To display the network as a rooted tree, the archaeological remains of the Liangzhu loom was selected as the ‘outgroup’, to define the root, since this is the earliest well-attested loom that has similarities with some of the contemporary looms in our sample. If we had not used the Liangzhu loom, the archaeological remains of a similar loom from the Dian culture (dated to around 2000 years ago) could have been used for this purpose, with similar results.

We carried out an analysis of ancestral states on the 70% consensus tree, calculating the ancestral states for all of the nodes, using a Maximum Likelihood model in Mesquite. We compared these ancestral states, and the character states of present-day looms with the character states of the deepest node in the tree, adjacent to the root, and calculated the number of gains and losses of characters at every node in the tree. We created a graphical representation of these gains and losses by superimposing them on the consensus tree (represented by the widths of connecting bars). This highlights the amount of change and differences in the degree of complexity that arose in each lineage. The procedure that we have used represents an approximation, since we have not calculated ancestral states on every tree in our original sample. We use the consensus tree for this reconstruction firstly because we put it forward as the ‘best hypothesis’ summarizing loom evolution in our dataset, and secondly since the ancestral states that are produced are mostly well-resolved and conform to what one would expect ‘by inspection’, ie by examining the characteristics of present-day looms and identifying shared features.

The interpretation of node ‘support values’

Numbers by each node in Fig 4 show the percentage of output trees that include that node. Such percentage values are often interpreted as ‘support values’, indicating the probability that a given split in the tree is correct. This may be a reasonable interpretation for datasets with significant amounts of missing data or uncertainty (‘noise’), but it is problematic in a case such as ours where the taxa (looms) are well characterized with few missing data points. In the case of our dataset we interpret node values of less than 100 as an indication of the presence of ‘conflicting’ signals that show that a unique tree-like model (involving strict vertical descent) cannot fully represent the development of looms at these points. Accordingly, we look for other processes such as hybridization or independent invention (phenomena that are classed as ‘data’, not ‘noise’) in order to fully explain the data in these instances.

Loom complexity versus societal complexity

We investigated the relationship between the complexity of looms and the complexity of the cultures that hosted them. To do this we used the number of distinct characters required to define each loom as a measure of complexity This is a conservative measure, since not all characters are equal in complexity: characters that are added later in loom development tend to consist of more complex and elaborate components.

We used data on political complexity from the Ethnographic Atlas [33] as a proxy for societal complexity. This data is available for around half of the looms/cultures in our survey. Despite being incomplete, it has the advantage of being independent of our loom evaluations, and therefore free from the possibility of researcher bias. The data are summarized in Supplementary Table 1. We made plots of loom complexity (expressed as the number of distinct characters required to describe each loom) versus political complexity (number of hierarchical levels in society as defined by Murdock) and evaluated these using simple statistical modelling (linear regression) using the statistical package R.

Supplementary Table 1: Political complexity (from Ethnographic Atlas) versus number of characters required to describe the loom.

| **Location/loom** | **Political complexity** | **Number of characters needed to describe the loom** |
| --- | --- | --- |
| Hainan Meifu Li | 2 | 38 |
| Hainan Qi Li | 2 | 39 |
| Hainan Ha Li | 2 | 39 |
| Taiwan Atayal | 1 | 34 |
| Nepal Sherpa | 2 | 43 |
| Tibetan | 4 | 48 |
| Alor Boka Besi | 2 | 38 |
| Flores Ili Mandiri | 2 | 40 |
| Sulawesi Toraja | 1 | 40 |
| Iban Kalimantan | 0 | 37 |
| Asho Chin | 1 | 36 |
| Zahau Chin | 1 | 35 |
| Toba Batak | 0 | 50 |
| Bali Karangsem | 2 | 50 |
| Timor Tetun | 1 | 37 |
| Phils Igorot | 0 | 36 |
| Micronesia Yap | 1 | 32 |
| Thailand Karen | 0 | 41 |
| Taiwan Yami | 0 | 37 |
| Malaysia Terengganu | 2 | 60 |
| Cambodia Kei thbanh | 3 | 47 |
| Hangzhou | 4 | 52 |
| Shanxi Dingcun | 4 | 54 |
| Guizhou Miao Panxian | 1 | 49 |
| Korea Hansan | 2 | 51 |
| Japan Maebashi | 4 | 49 |
| Ainu | 3 | 39 |
| Hangzhou Lesser Drawloom | 4 | 64 |
| Hangzhou Greater Drawloom | 4 | 63 |
| Sichuan Stepping Stone Loom | 4 | 48 |

**5. A model for the evolution of loom technology**

The Bayesian consensus tree, together with the ancestral forms (A, B, C, D, E, F, D, G, G1, G2) in Fig 4 and Fig 5 constitute a model for the development of the various looms that are found in East Asia today.

Based on the archaeological data described above, the earliest type of loom in Asia is known to be a foot-braced, back-tensioned type (node A). This type of loom persists today in a few, remote and widely scattered areas (Hainan, the Lao-Vietnam border region, upland areas in Taiwan), with very little apparent change.

The next development, chronologically, involved fixing the warp beam behind external supports (such as a couple of stakes in the ground), corresponding to the loom at node B. These developments allowed the weaver to weave a longer and wider cloth. Warp tensioning continued to be applied via a strap around the weaver’s back, attached to the cloth beam. The winding of the heddle changed from a spiral form to an alternating form, and a footrest was added. This loom design became extremely successful and is still widespread in Asia to this day, being found in many of the more remote islands in ISEA and upland regions of MSEA. Looms of this type consist of several clades (B1 to B6) that correspond to geographic regions, and some isolates. The clades are well resolved, but the phylogenetic relationships between them are rake-like, in other words there is no clear evolutionary hierarchy. The overall picture is of a fairly simple loom design, widely spread and subject to minor local variations, but with no great technological differences. The exception to this is a group of looms in clades B5 and B6 that have some more advanced features: a reed and (in some cases) a flat warping arrangement. The looms with these features do not form a clade (the Bayesian tree suggests that reed and flat warp are not ancestral). These technologies seem to have been ‘borrowed’ (hybridization) from more sophisticated frame looms in nearby Java and Sumatra, in which these features are ancestral. They allowed the owners of the simpler looms to weave more sophisticated prestige silk textiles decorated with supplementary weft designs (songket and similar types) more easily. These textiles were much in demand in this region, and this fact probably provided the driving force behind the horizontal transfer of these technologies.

The loom represented by node C constitutes a major technological advance. The ground-level loom at node B was transposed into a frame built around four uprights, incorporating a seat for the weaver. Instead of a circular warp (as in looms A and B) the warp is flat and wound onto the warp beam, allowing a much longer warp to be used and a correspondingly longer cloth to be woven (a length of 20 meters or more is common, as compared with 2-4 meters for types A and B). At around the same time a reed was added. This comb-like device spaces the warp yarns evenly, and helps to prevent tangling. At this early period the reed was not used for beating-in the weft. Despite these advances, the loom at node C remained body-tensioned, a feature inherited from older loom designs.

The addition of a rigid frame appears to have stimulated additional developments, which are represented by nodes D, E and F. These three nodes represent different solutions to the problem of allowing the weaver to raise the heddle by depressing a foot treadle or pulling on a cord, which left the weaver’s hands free for weft insertion. These developments speeded up the weaving process, and made it more convenient to add patterning. The presence of the frame provided the means to fix and support these heddle-raising arrangements. As noted above, archaeological data shows that these developments occurred before the Han period (before BCE 200)

In looms associated with node D, one or two treadles are attached to cords looped over supports above the weaver’s head, either on an overhead extension to the loom frame or the ceiling. This was ultimately the most successful of the three variants, giving rise to the majority of the more advanced frame looms used in Asia today. The earliest versions of these looms appear to have had a single heddle linked to a treadle (as in the ancestral loom at node D), adding a second heddle and treadle at node G. At this point the reed begins to be employed as a beater as well as a spacer. Because of their success, looms of this type became very widespread, particularly in the last two centuries, making their earlier distribution difficult to trace with certainty.

The loom at node E has the heddle attached to a short rocker that is attached to pillars positioned centrally in the loom frame. This type of loom appears to have been common in the Han dynasty (as attested by tomb murals from this period), and persists in a few rural looms today. These looms are mainly associated with Han Chinese speakers, or groups (such as the Tujia people) who have been closely associated with the Han.

The loom at node F represents yet another solution to the problem of raising the heddle. In this version a cord around the weaver’s foot passes around the back of the loom and connects to a long Y-shaped or U-shaped rocker beam, which is attached to the heddle at the end nearest to the weaver. As mentioned, this loom is also attested in Han dynasty archaeological remains. It is confined to rural areas today, but it has a very wide distribution, being found in Japan, southern and central China, southwest China and parts of MSEA, amongst a wide range of ethnicities. It does not appear to have spread into ISEA.

Complex patterning heddles, in which patterns were recorded on sticks (or cords) embedded in a matrix of long heddle cords, were developed by weavers in southwest China using looms descended from node F. These systems appear to have diffused ‘horizontally’ (hybridization) since they also occur in looms associated with node D, in which such patterning systems are not ancestral.

Additional references

Greenfield PM, Lave J 1982 Cognitive aspects of informal education. In: Cultural perspectives on child development. Wagner DA, Stevenson HW editors. WH Freeman, San Francisco CA.181-207.

Greenfield PM 1999 Cultural change and human development. New directions for child and adolescent development 83. doi: 10.1002/cd.23219998305

Feng Guangyu, editor 2006 Guangxi ethnic culture heritage collection 颐骆遗粹:广西百越文化文物精品集 China Social Sciences Press, Beijing.

Huson DH, Bryant D Application of Phylogenetic Networks in Evolutionary Studies. Mol. Biol. Evol. 2006 23(2) 254-267.

Maddison WP, Maddison DR 2011 Mesquite: a modular system for evolutionary analysis. Version 2.75 <http://mesquiteproject.org>

Vischer Michael P 1994 Black and red, white and yellow: socio-cosmic ideas in Palu’e textiles, in Hamilton RW (editor) Gift of the Cotton Maiden: Textiles from Flores and the Solor Islands. University of California 247-267.

Vollmer JE 1979 Archaeological evidence for looms from Yunnan, in I. Emery and P. Fiske (eds) Looms and their products: lrene Emery Roundtable on Museum Textiles 1977 Proceedings 78-89. Washington, DC, The Textile Museum.
