# Supplementary material for: The evolution of an ancient technology
Source: R Soc Open Sci. 2017 May 31;4(5):170208. doi: 10.1098/rsos.170208 (PMC5451833; doi:10.1098/rsos.170208)
Supplement: S2 Looms data sources [file rsos170208supp2.docx]

**Supplementary Material S2**

Looms survey: locations of looms and data sources, political complexity data from Ethnographic Atlas [33] (where available). Language names are from Ethnologue ([www.ethnologue.com](http://www.ethnologue.com)), accessed August 2016.

| Identifier | Language, dialect (Ethnologue) | Ethnographic Atlas code | Political complexity | Location | Loom type | Sources |
| --- | --- | --- | --- | --- | --- | --- |
| Liangzhu | unknown |  |  | Near present-day Hangzhou, China | Archaeological remains of a backstrap loom, dated to the Liangzhu culture (3400-2250BC) | [13,30] |
| Hainan Meifu Li | Hlai, Meifu |  |  | Donghezhen, Hainan Island | Foot-braced backstrap loom,  circular warp | [13] |
| Hainan Qi Li | Hlai, Qi |  |  | Shuimianqiao village, near Wuzhishan, Hainan Island | Foot-braced backstrap loom,  circular warp | [13] |
| Hainan Ha Li | Hlai, Ha |  |  | Ledong area, Hainan Island | Foot-braced backstrap loom,  circular warp | [13] |
| India Ladakh | Changthang |  |  | Rupshu area in Ladakh, | Woman’s backstrap loom, circular warp | [15] |
| Nepal Dolpo | Dolpo |  |  | Dho, Tarap valley, Nepal, backstrap loom | Backstrap loom, false-circular warp | Dunsmore 1993 |
| Nepal Chitre Magar | Magar |  |  | Chitre, Annapurna district, Nepal (Magar people) | Backstrap loom, circular warp | http://textiletrails.com/2012/09/29/ghada-techniques/#more-2300  accessed June 2014 |
| Nepal Annapurna Gurung | Gurung |  |  | Annapurna district, Nepal | Backstrap loom, circular warp | Dunsmore 1993: p142 onwards |
| India Apatani | Apatani |  |  | Lower Subansiri district, Arunachal Pradesh, India | Backstrap loom, circular warp | SOAS online photo archive http://digital.info.soas.ac.uk/cgi/gallery/59 accessed June 2015 |
| India Konyak Naga | Naga, Konyak |  |  | Nagaland (India and Myanmar) | Backstrap loom, circular warp | SOAS online photo archive http://digital.info.soas.ac.uk/cgi/gallery/58 accessed June 2015 |
| Myanmar Ashö Chin | Chin, Ashö | El19 | 1 | Northwestern Myanmar | Backstrap loom, circular warp | Fraser and Fraser 2005: Chapters 3 and 7 |
| Myanmar Zahau Chin | Chin, Falam, Zahau dialect | El19 | 1 | Northwestern Myanmar | Backstrap loom, circular warp | Fraser and Fraser 2005:Chapters 3 and 4 |
| Taiwan Atayal | Atayal |  |  | Northeastern Taiwan | Foot-braced backstrap loom, circular warp | Wu 1998, various online resources |
| Laos Katu FBBS | Katu, Western |  |  | Southern Laos, near border with Vietnam | Foot-braced backstrap loom, circular warp | Yoshimoto 2013: L8, various online resources |
| Taiwan Yami | Yami | Ia14 | 0 | Island off the Southwestern tip of Taiwan | Backstrap loom, circular warp | Yoshimoto 2013: L-36 |
| Philippines Igorot | Bontoc | Ia3 | 0 | Cordilleras, Luzon region, Philippines | Backstrap loom, circular warp | Yoshimoto 2013: L-19, various online resources |
| Philippines T’boli | Tboli |  |  | Mindanao | Backstrap loom, circular warp | Cooper-Cole 1913, 29, various online resources related to t’nalak weaving |
| Philippines Bagobo | Tagabawa |  |  | Mindanao | Backstrap loom, circular warp | Cooper-Cole 1913, Hamilton 1998, various online resources related to t’nalak weaving |
| Philippines B’laan | Blaan |  |  | Mindanao | Backstrap loom, circular warp | Cooper-Cole 1913, Hamilton 1998, various online resources related to t’nalak weaving |
| Philippines Mandaya | Mandaya |  |  | Mindanao | Backstrap loom, false circular warp | Cooper-Cole 1913 , Hamilton 1998, various online resources related to t’nalak weaving |
| Philippines Yakan | Yakan |  |  | Mindanao | Backstrap loom, false circular warp | various online resources |
| Micronesia Yap | Yapese | If6 | 1 | Yap, Micronesia | Backstrap loom, circular warp | photos online at http://digicoll.manoa.hawaii.edu  accessed June 2014 |
| Micronesia Solomon Islands | Sikaiana |  |  | Solomon Islands, Micronesia | Backstrap loom, circular warp | William Donner's Sikaiana Archive website http://www.sikaianaarchives.com/photos-2/loom-2/  accessed June 2014 |
| Thailand Karen | Karenic | Ei7 | 0 | Northern Thailand | Backstrap loom, circular warp | EB observations |
| Flores (West) | Manggarai |  |  | Flores, Indonesia | Backstrap loom, flat warp and reed | Gittinger 1979, Hamilton 1994 |
| Flores Sikka | Sika |  |  | Flores, Indonesia | Backstrap loom, circular warp | CB observations |
| Lamalera Lembata | Lamalera |  |  | Lamalera village, Lembata, Indonesia | Backstrap loom, circular warp | Hamilton 1994, CB observations |
| Lamalera Atadei | South Lembata |  |  | Atadei village, Lembata, Indonesia | Backstrap loom, circular warp | CB observations |
| Alor Boka Besi | Kui |  |  | Alor, Indonesia | Backstrap loom, circular warp | CB observations |
| Ternate Selatan | Retta |  |  | Selatan village, Ternate, Indonesia | Backstrap loom, circular warp | CB observations |
| Pantar Baranusa | Alor |  |  | Baranusa township, Pantar, Indonesia | Backstrap loom, circular warp | CB observations |
| Flores Ili Mandiri | Lamaholot, Ile Mandiri |  |  | villages in the Ili Mandiri region, East Flores, Indonesia | Backstrap loom, circular warp | CB observations |
| Timor Atoni | Uab Meto |  |  | West Timor, Indonesia | Backstrap loom, circular warp | Yaeger and Jacobsen 2002, CB observations |
| Timor Tetun | Tetun | Ic3 | 1 | Belu and regions to the west, West Timor, Indonesia | Backstrap loom, circular warp | Yaeger and Jacobsen 2002, CB observations |
| Sulawesi Toraja | Toraja, Sadan | Ic5 | 1 | To’Barana (northern part of Tana Toraja), Sulawesi, Indonesia | Backstrap loom, circular warp | Christou 1997 and 2004, CB observations. |
| Sulawesi Makassar | Makasar |  |  | Makassar, Sulawesi, Indonesia | Backstrap loom, flat warp and reed | Jasper and Pirngadie 1912-1930: 121, various internet resources |
| Kalimantan Iban | Iban | Ib1 | 0 | Ensaid Panjang, Kalimantan, Indonesia | Backstrap loom, circular warp | CB observations |
| Sumatra Palembang | Musi |  |  | Palembang area, Sumatra, Indonesia | Backstrap loom, flat warp and reed | CB observations |
| Toba Batak | Batak Toba | Ib4 | 0 | Lake Toba area, Sumatra, Indonesia | Backstrap loom, circular warp | Hamilton 1998 |
| Lombok | Sasak |  |  | Lombok, Indonesia | Backstrap loom, flat warp and reed | CB observations |
| Bali (Geringsing loom) | Bali | Ib3 | 2 | Tenganan Pegeringsingan, Bali, Indonesia | Backstrap loom, circular warp | Bühler et al 1975: Fig 9.2 p118, 30 |
| Bali Karangsem | Bali | Ib3 | 2 | Karangsem, Bali, Indonesia | Backstrap loom, flat warp and reed | Hauser-Schäublin et al 1991: p42, 35 |
| Guizhou Huishui Miao | Miao |  |  | Shangtian village, Jiarong district, Guizhou, China | Backstrap frame loom | [13] |
| Hunan Longshan Tujia | Tujia |  |  | Hunan, China | Backstrap frame loom | [13] |
| Hunan Jianghua Yao | Han, Cantonese (officially classified as Yao) |  |  | Jianghua region, Hunan, China | Backstrap frame loom | [13] |
| Guangxi Napo Zhuang | Zhuang, Yang |  |  | Napo region, Guangxi, China | Backstrap frame loom | [13] |
| Shanxi Dingcun | Han |  | 4 | Dingcun village, Shanxi, China | Backstrap frame loom | Online resources |
| Guizhou Zhaoxing Miao | Dong (officially classified as Miao) |  |  | Pingshan village, Zhaoxing district, Liping county, Guizhou, China | Frame loom | [13] |
| Yunnan Yingjiang Tai | Tai Nüa |  |  | Yingjiang county, Yunnan, China | Backstrap frame loom | Yoshimoto 2013: L-47 (incorrectly labeled ‘Bulan’), CB observations of Dai loom in Hangzhou Silk Weaving Museum |
| Yunnan Huayao Dai | Tai Ya |  |  | Yunnan, China | Backstrap frame loom | Yoshimoto 2013: L-46 |
| Guangxi Longji Yao | Bunu |  |  | Jinjiang village, Heping district, Longsheng county, Guangxi, China | Backstrap frame loom | [13] |
| Guizhou Antai Miao | Miao (some families have Han ancestry) |  |  | Antai district, Rongshui county, Guizhou, China | Backstrap frame loom | [13] |
| Guizhou Shidong Miao | Miao, Northern Qiandong |  |  | Mapo village, Taijiang county, Guizhou, China | Backstrap frame loom | [13] |
| Guizhou Bakai Miao | Miao, Southern Qiandong (Southern Hmu) |  |  | Bailai village, Bakai district, Rongjiang county, Guizhou | Backstrap frame loom | [13] |
| Guizhou Geyi Miao | Miao, Northern Qiandong |  |  | Nanyao village, Taipan township, Taijiang county, Eastern Guizhou,  China | Backstrap frame loom | [13] |
| Guizhou Panxian Miao | Miao, Small Flowery | Ed4 | 1 | Huashiban village, Machang district, Panxian county, Guizhou, China | Backstrap frame loom | [13] |
| Guizhou Zhaoxing Miao | Cao Miao (a Tai-Kadai group) |  |  | Guizhou, China | Frame loom | [13] |
| Korea Hansan | Korean | Ed1 | 2 | Hansan, Socheon county, Korea | Backstrap frame loom | UNESCO [online account](http://www.unesco.org/culture/ich/index.php?lg=en&pg=00011&RL=00453)  accessed June 2014 |
| Japan Maebashi | Japanese | Ed5 | 4 | Maebashi, Gunma prefecture, Japan | Backstrap frame loom | Yoshimoto 2013: L-138 |
| Japan Ainu | Ainu | Ec7 | 3 | Hokkaido, Japan | Backstrap frame loom | Yoshimoto 2013: L-129 |
| Hunan Tongdao Dong | Dong, Northern |  |  | Zhuanshui village, Boyang Nanmen district, Tongdao county, Hunan, China | Backstrap frame loom with compound pattern heddle | [13] |
| Guangxi Maonan | Maonan |  |  | Huanjiang county, Guangxi, China | Backstrap frame loom with compound pattern heddle | [13] |
| Guangxi Binyang Zhuang | Zhuang, Yongbei |  |  | Binyang county, Guangxi, China | Backstrap frame loom with compound pattern heddle | [13] |
| Guizhou Libo Buyi | Buyi |  |  | Kundi village, Jiarong county, Libo district, Guangxi, China | Backstrap frame loom with compound pattern heddle | [13] |
| Sichuan Stepping Stone | Han |  | 4 | Sichuan, China | Frame loom | CB observations of loom in Shanghai Textile Museum |
| Hangzhou Lesser Drawloom | Han |  | 4 | Hangzhou, China | Frame loom | CB observations of looms at the Hangzhou and Suzhou Silk Weaving Museums |
| Hangzhou Greater Drawloom | Han |  | 4 | Hangzhou, China | Frame loom | CB observations of looms at the Hangzhou and Suzhou Silk Weaving Museums |
| Hangzhou | Han |  | 4 | Hangzhou, China | Frame loom | CB observations loom at the Hangzhou Silk Weaving Museum, China |
| Nepal Sherpa Trestle | Sherpa |  |  | Nepal | Frame loom | Dunsmore 2009 |
| Tibet Trestle | Tibetan, Central |  |  | Tibet, China | Frame loom | Dunsmore 1993, CB observations |
| Guizhou Zhouxi Miao | Miao, Northern Qiandong (?) |  |  | Zhouxi township, Guizhou, China | Frame loom | [13] |
| Guizhou Miao Xijiang | Miao, Northern Qiandong |  |  | Xijiang village, Leshan county, Guizhou, China | Frame loom | CB observations |
| Guizhou Baiku Yao Yaoshan 2LH | lu Mien |  |  | Yaoshan, Guizhou, China | Frame loom | CB observations |
| Guizhou Buyi Xingyi | Buyi |  |  | Gaozhai village, Xingyi county, Guizhou, China | Frame loom | [13] |
| Guizhou Zhuang Jingxi | Zhuang, Yongnan |  |  | Jingxi region, Guangxi, China | Frame loom | [13] |
| Laos Xam Nuea Tai | Tai Daeng |  |  | Northern Laos | Frame loom | EB observations, Cheesman 2004 |
| Thailand Tai Isan | Thai, Northeastern or Phu Thai |  |  | Northern Thailand | Frame loom | EB observations at Baan Pon |
| Thailand Tai Yuan | Thai, Northern |  |  | Northern Thailand | Frame loom | CB observations of loom in the Chiang Mai Historical Museum |
| Vietnam Tai Dam | Tai Dam |  |  | Northern Vietnam | Frame loom | McClintock 2013, various internet resources |
| Vietnam Tai, single heddle | Tai Dam |  |  | Northern Vietnam | Frame loom | Deb McClintock - online journal http://www.mytripjournal.com/travel-684890  accessed April 2015 |
| Vietnam Pa Then | Pa-Hng |  |  | Tan Trinh, Northern Vietnam | Backstrap frame loom | Deb McClintock - online journal http://www.mytripjournal.com/travel-684890  accessed April 2015 |
| Sumatra Type I | Minangkabau |  |  | Minangkabau, Sumatra, Indonesia | Frame loom | Yoshimoto 1991: ‘Minangkabau Type I loom’ |
| Sumatra Type II | Minangkabau |  |  | Minangkabau, Sumatra, Indonesia | Frame loom | Yoshimoto 1991: ‘Minangkabau Type II loom’ |
| Malaysia Terengganu | Malay | Ej8 | 2 | Terengganu, Malaysia | Frame loom | Various online resources |
| Cambodia Kei thbanh | Khmer | Ej5 | 3 | Cambodia | Frame loom | Green 2003 |
| India Yongkham | Meitei |  |  | Manipur, India | Frame loom | Varadarajan and Amin-Patel 2008: 81-88 |

**Additional References**

Bühler A, Ramseyer U, Ramseyer-Gygi N 1975 Patola und gěringsing. Museum für Völkenkunde, Basel.

Cheesman P 2004 Lao-Tai textiles: the textiles of Xam Nuea and Muang Phuan. Studio Naenna, Chiang Mai.

Cooper-Cole F 1913 Wild tribes of the Davao district, Mindanao. <http://www.gutenberg.org/files/18273/18273-h/18273-h.htm>

Christou M 1997 An ethnographic study of the loom and weaving of the Sa'dan Toraja of Tobaranna. Universty of Alberta - National Library of Canada.

Christou M 2004 Sa’dan Toraja supplementary weft weaving: an ethnographic interpretation of acculturation and assimilation of loom technology and weaving techniques. Textile Society of America Symposium Proceedings Paper 485. http://digitalcommons.unl.edu/tsaconf/485

Dunsmore S 1993 Nepalese Textiles. British Museum Press, London.

Fraser DW, Fraser BG 2005 Mantles of merit: Chin textiles from Myanmar, India and Bangladesh. River Books, Bangkok.

Gittinger M 1979 Splendid symbols: textiles and tradition in Indonesia. Textile Museum, Washington DC.

Green G 2003 Traditional textiles of Cambodia. River Books, Bangkok.

Hauser-Schäublin B, Nabholz-Kartaschoff M, Ramseyer U 1991 Balinese textiles. Periplus, Singapore.

Jasper JE, Pirngadie M 1912-1930 De inlandsche kunstnijverheid in Nederlandsch Indië. 's-Gravenhage, Mouton.

Hamilton RW editor 1994 Gift of the cotton maiden: textiles from Flores and the Solor Islands. University of California.

Hamilton RW editor 1998 From the rainbow's varied hue: textiles of the southern Philippines. UCLA Fowler Museum, Los Angeles.

Mc Clintock D 2013 Storing pattern above the warp line. Complex Weaver’s Journal, June 2013: 5-7.

Varadarajan L, Amin-Patel K 2008 Of fibre and loom. National Institute of Design, Ahmedabad.

Wu Shiu-Hui 1998 The characteristics of Taiyal weaving as an art form. Durham theses, Durham University. Durham E-Theses Online: <http://etheses.dur.ac.uk/5054/>

Yaeger RM, Jacobson MI 2002 Textiles of western Timor. White Lotus Press, Bangkok.

Yoshimoto S 1991 Typological studies of Indonesian Handlooms (1) types and distribution. Bulletin of the National Museum of Ethnology Vol 15, 1 国立民族学博物館研究報告15巻1号.

Yoshimoto S 2013 Textiles and looms of the world 吉本忍,世界の織機と織物 L128, L129. Minpaku Museum, Japan. 国立民族学博物館 編.
